# Spatiotemporal differentiation and coupling mechanisms between vegetation and ecological water demand in a typical arid region of Xinjiang, China

**DOI:** 10.3389/fpls.2026.1913154

**Published:** 2026-08-06

**Authors:** Qi An, Yang Wang, Xiang Li, Chun Yi

**Affiliations:** 1 Key Laboratory of Ministry of Education of Grassland Resources and Ecology in Western Arid Region, College of Grassland Science, Xinjiang Agricultural University, Xinjiang, China; 2 College of Resources and Environment, Xinjiang Agricultural University, Xinjiang, China

**Keywords:** NDVI–EWD relationship, phenological stages, coupling coordination degree, driving mechanisms, water resource constraints, ecological restoration

## Abstract

**Introduction:**

Climate change and anthropogenic activities have jointly reshaped vegetation growth processes and water-consumption patterns in arid regions, complicating the relationship between vegetation restoration and water-resource constraints. Existing studies have mainly focused on individual ecological processes. A systematic understanding of how vegetation growth is coupled with ecological water demand (EWD), and of the mechanisms driving this relationship, remains limited.

**Methods:**

Xinjiang was selected as a representative arid region, and multi-source datasets from 2000 to 2024 were used. The vegetation growing season was divided into the start of season (SOS), peak of season (POS), and end of season (EOS). Trend analysis, the coupling coordination degree (CCD) model, quadrant analysis, GeoDetector, and PLS-SEM were applied to characterize the spatiotemporal patterns of the Normalized Difference Vegetation Index (NDVI) and EWD, identify their coupling types, and reveal the driving mechanisms of NDVI–EWD coupling.

**Results and discussion:**

The results show that: (1) The NDVI and EWD of Xinjiang showed fluctuating upward trends from 2000 to 2024. The improvement of vegetation was mainly concentrated in the northern and southern slopes of the Tianshan Mountains, the Ili River Valley, the southern margin of the Altai Mountains and the periphery of the Tarim Basin. The highest EWD occurred in the POS stage, reaching 139.90 mm. (2) The CCD between NDVI and EWD generally shifted from imbalance toward coordination. Areas characterized by low vegetation cover and limited EWD (LL) remained the dominant coupling type, but their extent continued to decrease. The main transition was toward areas with high vegetation cover and limited EWD (HL). The most active type conversion occurred during the POS stage. (3) Land-use intensity, topographic wetness index, evapotranspiration, temperature and soil organic carbon were identified as important driving factors, and climate, topography and anthropogenic activities affected the coupling intensity through the soil environment, water redistribution and land-use processes. The findings provide scientific support for the assessment of vegetation restoration, the optimization of ecological water-supplementation and the management of water resource carrying capacity in arid regions.

## 1 Introduction

Global climate change and human disturbance have a major impact on the structure and function of terrestrial ecosystems (Parmesan et al., 2022; Zhang et al., 2022; Higgins et al., 2023), and the relationship between vegetation growth, hydrological processes and ecosystem stability is receiving increasing attention. Vegetation is an important component of terrestrial ecosystems, which directly reflects the combined effect of climate change and human disturbance. It also plays an important role in soil and water conservation, regulation of carbon cycle and maintenance of ecological security (Piao et al., 2019; Xiong et al., 2024). There is a general greening trend of global vegetation in the last decades. However, increased vegetation cover does not necessarily indicate a corresponding improvement in ecosystem stability (Wang et al., 2023c). Vegetation restoration or increase in vegetation cover in arid regions can affect regional evapotranspiration and water-consumption patterns, which may impose additional pressure on limited water resources (Jia et al., 2024; Zhu et al., 2025) A better understanding of this vegetation–water linkage can help explain ecosystem change and facilitate water-resource regulation in these regions.

Drylands cover approximately 41.5% of the earth’s land surface and are sensitive to climate change and human disturbance, with ecosystems generally fragile (Wang et al., 2023a). The sparse precipitation and high evaporative demand make water availability the primary limiting factor for vegetation growth and ecosystem productivity in drylands. Ecological water demand (EWD) refers to the amount of water required to sustain normal vegetation growth, community stability and basic ecosystem functions (Huang et al., 2024). EWD is an important index reflecting vegetation growth’s dependence on water resources. EWD has been widely applied in water-resource allocation, ecological restoration, and water-stress assessment in arid inland river basins (Hao et al., 2023; Wang et al., 2023b). However, EWD varies with climatic evaporative demand, vegetation type, growth stage, soil moisture conditions, and human disturbance. Vegetation restoration may therefore become mismatched with available water resources. Examining the coupling between vegetation growth and EWD can help identify water-matching patterns and potential mismatch risks during vegetation restoration in arid regions.

Remote sensing provides extensive spatial coverage, frequent observations and high data quality, which is a useful tool to monitor vegetation growth and ecohydrological processes at the regional scale (de Araujo Barbosa et al., 2015; Kooistra et al., 2024; Mekonnen et al., 2025). NDVI is among the most common remote sensing indices to illustrate the extent, activity, and spatiotemporal variation of vegetation health (Huang et al., 2020). It has been extensively used in the observation of changes of ecosystems and vegetation responses to environmental factors. Compared with NDVI that mainly represents the state of vegetation, EWD provides information on the water needed to sustain vegetation growth and ecosystem functions. EWD is commonly estimated from reference evapotranspiration, vegetation coefficients, and water-stress coefficients under the FAO-56 framework for reference evapotranspiration and crop coefficient estimation (Allen et al., 1998). In arid regions, vegetation growth is strongly constrained by water stress. The soil-water stress coefficient (Ks) introduced into the EWD estimation improves the description of the vegetation water demand and the degree of soil moisture limitation on this demand. This method has been applied to the estimation of EWD, assessment of water stress and water-resource management in arid areas (Hao et al., 2023; Wang et al., 2023b). Previous studies have focused on the analysis of NDVI dynamics, EWD estimation and the driving factors of their change, but most of the studies analyzed the change of vegetation and ecological water demand as separate processes, mainly considering the spatiotemporal variation, trend features or influencing factors of single indicators (Huang et al., 2024; Mehmood et al., 2024; Lu et al., 2025). Less attention has been paid to how vegetation growth and EWD are spatially matched or mismatched, especially across different phenological stages. This gap is important in arid regions, where vegetation growth is strongly seasonal and water demand may be controlled by different environmental factors at the start, peak, and end of the growing season. Therefore, the NDVI–EWD coupling relationship needs to be examined across phenological stages.

Xinjiang lies in the interior of Eurasia and is an important part of the Central Asian drylands. It is also one of the major arid regions in northwestern China. Mountains, oases, and deserts occur in close proximity across the region. Water resources are highly uneven in space and time, and vegetation growth is strongly tied to water availability (Mo et al., 2019; Fan et al., 2020; Yao et al., 2022). In recent years, regional warming and wetting, oasis expansion, ecological restoration, and land-use change have further complicated the relationship between vegetation-cover change and EWD. In this study, Xinjiang was used as a representative arid region to examine the coupling between vegetation growth and EWD. A multi-source data framework was developed to characterize the spatiotemporal patterns of NDVI and EWD and to evaluate their coupling relationship across phenological stages. The objectives were to: (1) Characterize the spatiotemporal patterns and trends of NDVI and EWD during different phenological stages; (2) Identify spatial matching and mismatch patterns between NDVI and EWD and classify their coupling coordination types; and (3) Examine how climate, topography, soil conditions, and anthropogenic activities influence the spatial differentiation of NDVI–EWD coupling. This study provides evidence to support vegetation restoration, water-resource allocation, and ecological security management in arid regions.

## 2 Materials and methods

**Figure 1:**
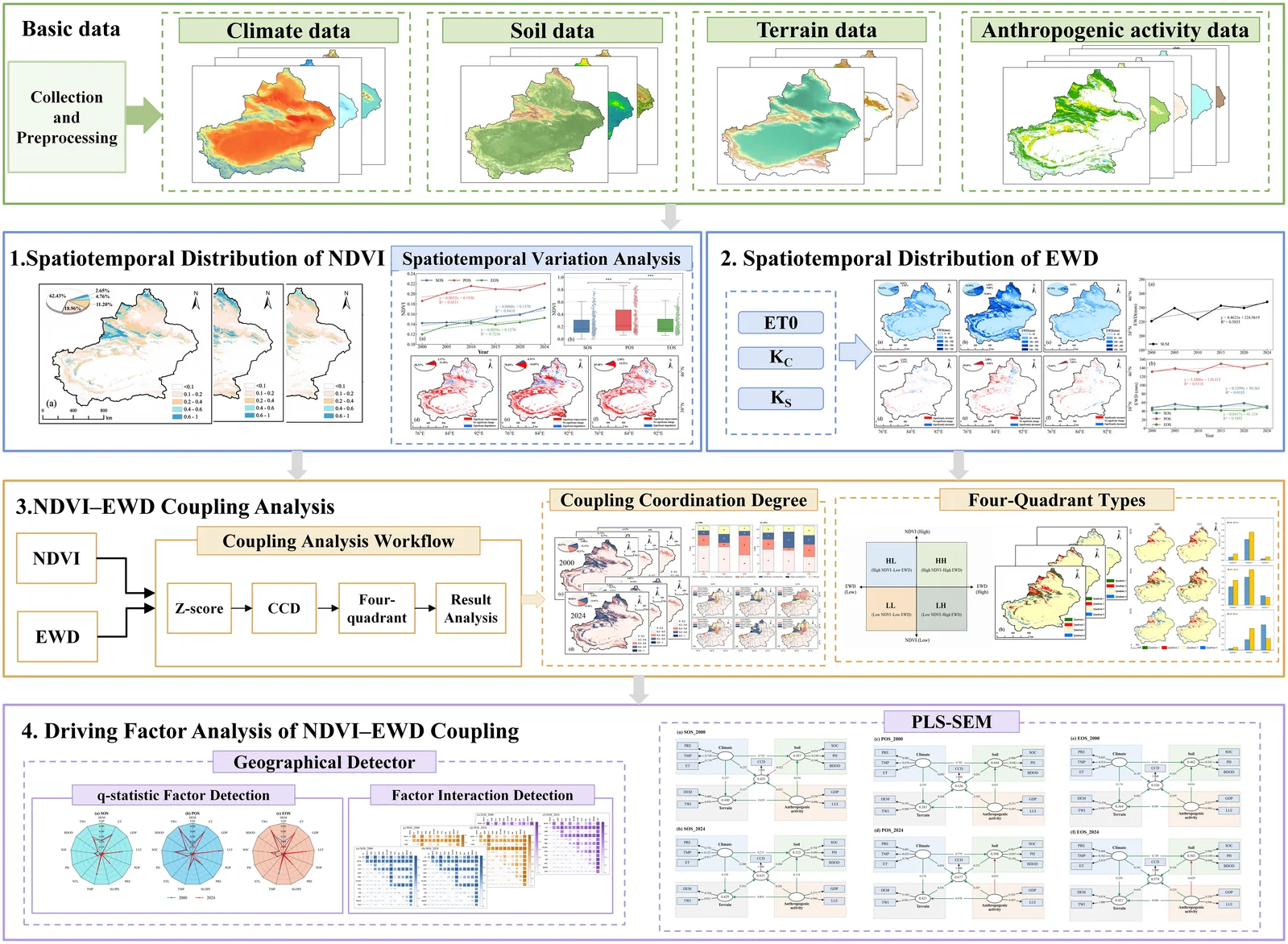
Research framework of this study.

An integrated research framework was developed to analyze the coupling between NDVI and EWD in the arid region of Xinjiang (**Figure 1**). The framework consisted of data collection and preprocessing, quantitative assessment of the spatiotemporal patterns of NDVI and EWD, CCD and quadrant analyses, and identification of driving factors and their mechanistic pathways. Based on this framework, type-specific management and regulation strategies were proposed.

### 2.1 Study area

**Figure 2:**
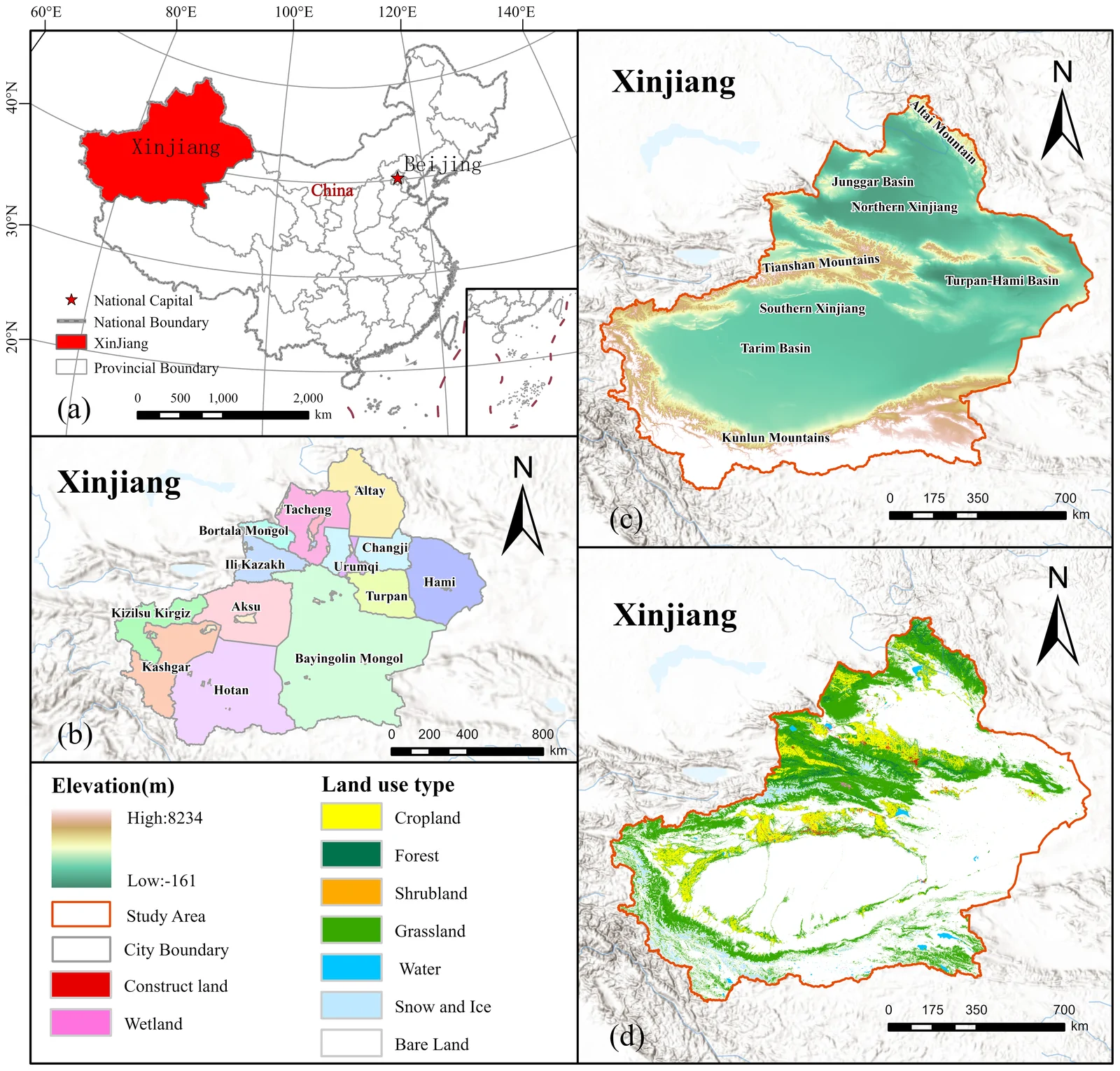
Overview of the study area. **(a)** Geographical location of Xinjiang. **(b)** county administrative boundaries. **(c)** spatial distribution of elevation. **(d)** land-use pattern in 2024.

Xinjiang was selected as the study area (**Figure 2**). It is located in arid northwestern China, deep in the interior of Eurasia, and covers approximately 1.6649 million km². The region is characterized by the well-known “three mountains and two basins” geomorphic pattern. The Junggar Basin lies between the Altai Mountains and the Tianshan Mountains, whereas the Tarim Basin lies between the Tianshan Mountains and the Kunlun Mountains. This landform structure has shaped a typical mountain–oasis–desert ecosystem. Xinjiang has a temperate continental arid climate. Precipitation is limited, evaporation is strong, and the long-term mean annual precipitation is generally less than 200 mm. Under the combined influence of topography and water limitation, clear hydrothermal gradients have developed among mountains, basins, and oases. Deserts, grasslands, and croplands are the dominant ecosystem types in the region. Vegetation dynamics are therefore highly sensitive to changes in hydrothermal conditions (Zhang et al., 2024a; Lu et al., 2025). These environmental characteristics make Xinjiang a suitable region for examining vegetation dynamics, EWD, and their coupling mechanisms.

### 2.2 Data sources

Remote sensing, climate, soil, topographic, and human activity data were integrated to construct a multi-source dataset for characterizing vegetation change, EWD, and their driving factors in Xinjiang (**Table 1**). To ensure spatial consistency across datasets with different sources and resolutions, all data were projected to the CGCS2000 GK Zone 16 coordinate system and resampled to a spatial resolution of 1 km. For spatial continuity, continuous variables (e.g., NDVI, temperature, precipitation, evapotranspiration, soil properties, DEM and slope) were resampled with bilinear interpolation, and categorical variables (e.g., land-use data) were resampled with the nearest neighbor method to preserve the original class attributes and avoid the generation of artificial land-use categories. This preprocessing procedure ensured spatial consistency among datasets, and maintained the data characteristics of different variable types. Specifically, NDVI was derived from the MODIS MOD13Q1 product, EWD estimation was based on potential evapotranspiration, land-use data, ERA5-Land meteorological data, SoilGrids soil-property data, and MODIS land surface temperature data. The driving factors were grouped into four categories: climate, soil, terrain, and anthropogenic activity. Climate factors included precipitation (PRE), temperature (TMP), and evapotranspiration (ET); Soil factors included soil organic carbon (SOC), soil pH (pH), and soil bulk density (BDOD); Terrain factors included elevation derived from the digital elevation model (DEM), topographic wetness index (TWI), and slope; Anthropogenic activity factors included gross domestic product (GDP), land-use intensity (LUI), population (POP), and nighttime lights (NTL).

**Table 1:** Datasets and sources.

Data type	Data	Resolution	Time	Source
NDVI	MODIS MOD13Q1	250 m	2000–2024	Google Earth Engine (https://code.earthengine.google.com)
EWD	Potential Evapotranspiration (PET)	1 km	2000–2024	Terra Climate (https://www.climatologylab.org/terraclimate.html)
	Land Use/Land Cover (LULC)	30 m	2000–2024	Zenodo (https://zenodo.org)
	ERA5-Land Hourly	0.1°×0.1°	2000–2024	Climate Data Store (https://cds.climate.copernicus.eu/)
	SoilGrids	250 m		International Soil Reference and Information Centre (https://data.isric.org/)
	MODIS Land Surface Temperature (LST)	1 km	2000–2024	National Aeronautics and Space Administration (https://modis.gsfc.nasa.gov/)
Climate	Precipitation (PRE)	1 km	2000–2024	National Tibetan Plateau Data Center (https://data.tpdc.ac.cn/)
	Temperature (TMP)			
	Evapotranspiration (ET)	10 km	2000–2024	Global Land Evaporation Amsterdam Model (https://www.gleam.eu/)
Soil	Soil Organic Carbon (SOC)	250 m/ 1 km		International Soil Reference and Information Centre (https://data.isric.org/)
	Soil pH (PH)			
	Bulk Density of Oven-dried soil (BDOD)			
Terrain	Digital Elevation Model (DEM)	1 km		National Earth System Science Data Center (https://www.geodata.cn)
	Topographic Wetness Index (TWI)			
	Slope			
Anthropogenic activity	Gross Domestic Product (GDP)		2000-2023	Resource and environment science and data center (https://www.resdc.cn/)
	Land Use Intensity (LUI)	30 m	2000–2024	Zenodo (https://zenodo.org)
	Population (POP)	1 km	2000-2025	Global Human Settlement Layer (https://human-settlement.emergency.copernicus.eu/)
	Nighttime Lights (NTL)	1 km	2000–2024	Harvard Dataverse (https://dataverse.org/)

NDVI, EWD, and related climate variables were extracted and aggregated for three phenological stages: the start of season (SOS, March–May), peak of season (POS, June–August), and end of season (EOS, September–October). This allowed all dynamic variables to be analyzed within the same phenological windows. This fixed-window division is consistent with previous seasonal NDVI studies in Xinjiang and Central Asia, where vegetation dynamics have commonly been analyzed using spring, summer, and autumn windows based on seasonal variations in NDVI and climate conditions (Zhang et al., 2021; Lu et al., 2025). Relatively stable background variables, including SOC, pH, BDOD, DEM, TWI, and slope, were treated as static variables in the subsequent analyses. Where dataset temporal coverage was not complete, data from surrounding years were used to ensure temporal continuity and to reduce the impact of missing observations.

### 2.3 Methods

#### 2.3.1 Normalized difference vegetation index

NDVI is a widely used remote sensing-based vegetation monitoring index, providing a useful indication of vegetation greenness, vegetation cover and spatiotemporal vegetation dynamics (Huang et al., 2020). In this study, the MODIS MOD13Q1 data were processed on the Google Earth Engine (GEE) platform to generate NDVI raster datasets for different phenological stages from 2000 to 2024. NDVI was calculated as shown in Equation 1:

(1) $$NDVI=\frac{\rho_{NIR}-\rho_{Red}}{\rho_{NIR}+\rho_{Red}}$$

where NIR and Red denote the near-infrared and red reflectance bands. The theoretical range of NDVI ∈ [−1, 1]. Higher NDVI values generally indicate greater vegetation cover and better vegetation conditions. In this study, NDVI was grouped into five classes: I ≤ 0.1; 0.1< II ≤ 0.2; 0.2< III ≤ 0.4; 0.4< IV ≤ 0.6; 0.6< V ≤ 1.

#### 2.3.2 Ecological water demand of vegetation

EWD is the water demand for normal vegetation growth and basic ecosystem functions under specific climatic, land surface, and soil moisture conditions. In the FAO-56 framework, the atmospheric demand for evaporation was represented by the reference evapotranspiration (ET_0_), and the vegetation coefficient (Kc) was used to compensate the effects of vegetation type, canopy cover, and growth stage. This method has been widely used to estimate ecosystem evapotranspiration and vegetation water demand (Allen et al., 1998; Pereira et al., 2024). The soil moisture limitation coefficient (Ks) was introduced to compensate the EWD estimates at different phenological stages in Xinjiang due to the severe soil moisture stress to limit the vegetation water use in arid and semi-arid regions (D’Odorico et al., 2009; Wang et al., 2023b). The EWD was calculated as shown in Equations 2, 3:

(2) $${EW}_{P}=ET_{0}\times K_{S}\times K_{C}$$

(3) $$EWD=\sum_{P=1}^{N}{EW}_{P}\times A_{P}$$

where EW_P_ is the EWD quota (mm); ET_0_ is the reference evapotranspiration (mm); Kc is the vegetation coefficient; Ks is the soil moisture limitation coefficient; EWD is the EWD of vegetation (mm); Ap is the area of a grid cell (km²); N is the total number of grid cells in the study area.

Reference ET_0_ was estimated using the FAO-56 Penman–Monteith equation (Miralles et al., 2025), as shown in Equation 4:

(4) $${ET}_{0}=\frac{0.408\Delta \left(R_{n}-G\right)+\gamma \frac{900}{T+273}u_{2}\left(e_{s}-e_{a}\right)}{\Delta +\gamma \left(1+0.34u_{2}\right)}$$

where Δ is the slope of the saturation vapor pressure curve (kPa °C^-^¹); Rn is the net radiation at the crop surface (MJ m^-^² d^-^¹); G is the soil heat flux density (MJ m^-^² d^-^¹); u is the mean wind speed at a height of 2 m (m s^-^¹); e_s_ is the saturation vapor pressure (kPa); e_a_ is the actual vapor pressure (kPa); γ is the psychrometric constant (kPa °C^-^¹); T is the mean air temperature at a height of 2 m (°C).

In this study, TVDI was used to derive the soil moisture limitation coefficient, because it provides a relative measure of surface moisture conditions based on the NDVI–LST feature space (Sandholt et al., 2002; Li et al., 2023). TVDI is usually negatively correlated with measured relative soil moisture, and its values generally range from 0 to 1, with higher values indicating drier conditions. Because the FAO-56 soil water stress coefficient Ks is also bounded between 0 and 1, Ks was defined as 1 − TVDI and interpreted as a TVDI-derived relative wetness coefficient, which was used to represent pixel-scale soil moisture availability. To derive TVDI and estimate Ks, MOD13Q1 NDVI and MOD11A2 land surface temperature (LST) products were used to construct the NDVI–LST feature space on the GEE platform, and wet and dry edges were extracted to establish the dry–wet triangle for characterizing soil moisture conditions across the study area.

To ensure consistency in TVDI construction (Equation 7), MOD13Q1 NDVI was resampled to the same 1 km spatial resolution of MOD11A2. Specifically, NDVI values within the study area were grouped into bins at an interval of 0.01, and the minimum LST value (T_s,min_) and maximum LST value (T_s,max_) were calculated for each NDVI bin. T_s,min_ and T_s,max_ were taken as observations of the wet and dry edges. Linear least-squares regressions were fitted using NDVI as the independent variable and T_s,min_ and T_s,max_ as dependent variables, from which the empirical relationships for the wet and dry edges were obtained as shown in Equations 5, 6:

(5) $$T_{s,min}(NDVI)=a_{min}\cdot NDVI+b_{min}$$

(6) $$T_{s,max}(NDVI)=a_{max}\cdot NDVI+b_{max}$$

(7) $$TVDI=\frac{T_{s}-T_{s,min}(NDVI)}{T_{s,max}(NDVI)-T_{s,min}(NDVI)}$$

where Ts is the average land surface temperature of the pixel. TVDI ranges from 0 to 1. A higher TVDI value indicates that the pixel is closer to the dry edge in the NDVI–LST triangle and therefore represents drier surface conditions. Conversely, a lower TVDI value indicates that the pixel is closer to the wet edge and has better soil moisture conditions.

A multi-year, multi-phenological-stage workflow was developed on the GEE platform to estimate the vegetation coefficient (Kc). Vegetation cover from remote sensing, vegetation height derived from land-use and ERA5-Land meteorological variables were combined to generate spatially explicit estimates of Kc using the FAO-56 crop coefficient methodology (Allen and Pereira, 2009). Kc was calculated as shown in Equations 8, 9:

(8) $$K_{\mathrm{c,}}=K_{c,min}+\left(K_{c,full}-K_{c,min}\right)\left\{min\left[1,2f_{p},{\left(f_{\text{p}eff}\right)}^{\left(\frac{1}{1+h}\right)}\right]\right\}$$

(9) $$K_{c,ful}=K_{c,h}+\left[\begin{array}{c} 0.04(u_{2}-2)-0.004({RH}_{min}-45) \end{array}\right]{\left(\frac{h}{3}\right)}^{0.3}$$

where K_c,min_ is the minimum vegetation coefficient; K_c,full_ is the vegetation coefficient under full-cover conditions; f_p_ is the effective vegetation cover; h is the mean vegetation height during different phenological stages; K_c,h_ is the baseline coefficient under high-cover conditions; u_2_ is the wind speed at a height of 2 m; RH_min_ is the minimum relative humidity.

#### 2.3.3 Sen’s slope and Mann–Kendall trend test

To detect the long-term trends of NDVI and EWD in different phenological stages, the Sen’s slope estimator (Sen, 1968) was combined with the Mann–Kendall (MK) nonparametric trend test (Mann, 1945). Pixel-by-pixel trend analysis was performed for the EWD raster datasets during the SOS, POS, and EOS stages from 2000 to 2024. Sen is a robust trend estimator based on the median of pairwise slopes. It reduces the influence of outliers on the estimated trend magnitude. The MK test is a nonparametric method widely used to detect monotonic trends in time-series data. It is suitable for ecological, hydrological, climatic and remote sensing time series analysis (Liu et al., 2025). The equations are as follows:

For a time series X_1_, X_2_…, X_n_, with a length of n, Sen’s slope was calculated as shown in Equation 10:

(10) $$\beta =Median\left(\frac{x_{j}-x_{i}}{j-i}\right),1\leq i<j\leq n$$

where β is the trend slope of the time series. X_i_ and X_j_ denote the variable values in years i and j, i, j ∈ [2000, 2024]. When β>0, the study variable shows an increasing trend during the study period. When β<0, it shows a decreasing trend. A value of β equal to or close to 0 indicates no clear trend.

The MK test was applied to determine whether a significant monotonic increasing or decreasing trend was present in the time series. The test statistic S was calculated as shown in Equations 11, 12:

(11) $$S=\sum_{i=1}^{n-1}\sum_{j=i+1}^{n}sgn(x_{j}-x_{i})$$

(12) $$sgn(x_{j}-x_{i})=\{\begin{array}{c} 1,x_{j}-x_{i}>0\\ 0,x_{j}-x_{i}=0\\ -1,x_{j}-x_{i}<0 \end{array}$$

where X_i_ and X_j_ denote the observed values in years i and j, with i, j ∈ [2000, 2024]. n is the length of the time series. In this study, n = 25. When S > 0, the time series shows an overall increasing trend. When S< 0, it shows a general decreasing trend. A value of S close to 0 indicates no clear trend. For large sample sizes, the statistic S approximately follows a normal distribution, and its variance can be expressed as shown in Equation 13:

(13) $$Var(S)=\frac{n(n-1)(2n+5)}{18}$$

The standardized test statistic Z was calculated as shown in Equation 14:

(14) $$Z=\{\begin{array}{c} \frac{S-1}{\sqrt{Var(S)}},S>0\\ 0,S=0\\ \frac{S+1}{\sqrt{Var(S)}},S<0 \end{array}$$

The significance of the trend was determined using the critical values of the standard normal distribution. The trend is significant at the 95% confidence level if |Z| > 1.96 at α= 0.05. The trend is significant at the 99% confidence level if |Z| > 2.58 at α= 0.05. A positive Z value indicates an increasing trend, while a negative Z value indicates a decreasing trend. The trend was classified as significant or nonsignificant based on the corresponding |Z| threshold.

#### 2.3.4 Coupling coordination degree model (CCD)

To reveal the interaction between the two elements and their level of coordinated development, the CCD model was used for quantitative analysis. Previous studies have shown that this model can be used to quantify interactions among different systems and to evaluate their degree of coordinated development. This model has been widely applied in assessments of resources, ecological environments, and regional sustainable development (Gao, 2024; Kang et al., 2024). In this study, vegetation growth status and EWD were treated as two subsystems. The subsystem indices U_1_ and U_2_ were constructed, and the coupling degree, comprehensive evaluation index, and CCD were calculated after normalization. The calculation formulas are as shown in Equations 15–17:

(15) $$C=\frac{2\sqrt{U_{1}U_{2}}}{U_{1}+U_{2}}$$

(16) $$T=\alpha U_{1}+\beta U_{2}$$

(17) $$D=\sqrt{C\times T}$$

where C is the coupling degree, and U_1_ and U_2_ denote the normalized subsystem indices; T represents the comprehensive development level of the two components. α and β are the weights assigned to U_1_ and U_2_, with α + β = 1. In this study, vegetation growth status and EWD were considered equally important because both jointly characterize the vegetation–water relationship in regional ecological processes, α = β = 0.5; D denotes the CCD. A larger D value indicates a higher degree of coordination between the two components, and smaller D value indicates weaker coordination.

The threshold intervals were determined according to the previous researches (Dong et al., 2019; Li et al., 2021) and the continuous CCD was classified into different types of coordination. This classification followed the concept of membership intervals in fuzzy mathematics. A grading standard for the coupling coordination level between NDVI and EWD was established for the study area (**Table 2**). The county-level classification was derived using zonal statistics and a dominant-type rule based on proportions. The pixel-level CCD classes and quadrant types were overlaid on the county administrative boundaries to determine the area proportions of each type within each county. We used the majority rule principle as is common in categorical spatial aggregation (Gann, 2019) and the logic of mixed-type classification (Yang et al., 2024) that if the largest type occupied ≥50% of the county area, the county was classified into the dominant type; or if the largest type occupied<50% of the county area but the difference between the largest and second largest types was ≥10%, the county was classified into the largest type.

**Table 2:** Coupling coordination degree level.

Coupling coordination level	Value range	Meaning
1	CCD ∈ (0.801,1.000]	High coordination
2	CCD ∈ (0.601,0.800]	Moderate coordination
3	CCD ∈ (0.401,0.600]	Basic coordination
4	CCD ∈ (0.201,0.400]	Moderate imbalance
5	CCD ∈ (0.000,0.200]	Serious imbalance

#### 2.3.5 Four-quadrant model

To further identify the spatial matching relationship between vegetation growth and EWD, a four-quadrant classification method was introduced in this study. This method classifies the joint state of two indicators based on the cross-relationship between the two indicators. The relationship between two variables can be classified into four categories based on the high-low combination of the two variables: high-high, high-low, low-low and low-high (Kou et al., 2024; Zhang et al., 2024c). This classification can directly describe the spatial matching, mismatch and coordination of different factors. In this study, the four-quadrant model was used to analyze the spatial combination patterns between NDVI and EWD. EWD was set as the x-axis, and NDVI was set as the y-axis. The threshold of 0.5 was used to distinguish high and low values. This midpoint threshold has been used in previous four-quadrant studies based on normalized ecological indicators to distinguish high and low categories and to identify different coupling types (Sun et al., 2020; Guo et al., 2025). The first quadrant represented the areas with high NDVI and high EWD. The second quadrant represented the areas with high NDVI and low EWD. The third quadrant represented the areas with low NDVI and low EWD. The fourth quadrant represented the areas with limited NDVI and high EWD.

#### 2.3.6 Centroid migration method

Centroid migration analysis was used to describe the spatial displacement of different NDVI–EWD quadrant coupling types during the study period. This method has been widely used to characterize the migration direction and distance of different spatial types in land-use and landscape-pattern studies (Fan et al., 2025). For each quadrant type, the centroid coordinates were calculated based on the area-weighted spatial distribution of all pixels belonging to that type, as shown in Equations 18, 19:

(18) $$X_{t}=\frac{\sum_{i=1}^{n}A_{i}x_{i}}{\sum_{i=1}^{n}A_{i}}$$

(19) $$Y_{t}=\frac{\sum_{i=1}^{n}A_{i}y_{i}}{\sum_{i=1}^{n}A_{i}}$$

where X_t_ and Y_t_ represent the centroid coordinates of a given coupling type in year t; x_i_ and y_i_ are the coordinates of pixel i; A_i_ is the area of pixel i; and n is the number of pixels belonging to the same coupling type. Since all raster datasets were resampled to the same spatial resolution, A_i_ was constant for all pixels. The migration distance between two periods was calculated as shown in Equation 20:

(20) $$D=\sqrt{{\left(X_{t2}-X_{t1}\right)}^{2}+{\left(Y_{t2}-Y_{t1}\right)}^{2}}$$

where D is the migration distance, and X_t1_, Y_t1_ and X_t2_, Y_t2_ are the centroid coordinates in two different years. The centroid migration direction and distance were used to indicate the spatial reorganization of NDVI–EWD coupling types from 2000 to 2024.

#### 2.3.7 GeoDetector

The GeoDetector is a statistical method to detect spatial stratified heterogeneity and its influencing factors (Wang et al., 2016). The core principle is to test whether the spatial stratification of the explanatory variable X can explain the spatial differentiation of the dependent variable Y. The explanatory power of the factors is quantified by the q value, q∈ [0,1]. The higher the q value, the more significant the explanatory power of spatial differentiation (He and Liu, 2024; Ke et al., 2025). NDVI and EWD CCD were taken as dependent variables, climate, topography, soil, and human activity factors were selected as explanatory variables. The factor detector was used to quantify the explanatory power of individual factors, and the interaction detector was used to determine whether the combined effects of two factors were enhanced or weakened. This method has been widely applied in vegetation and ecological driving-factor studies (Xia et al., 2024; Sun et al., 2025). In this study, this method was used to explore the joint effects of different factors on the coupling between vegetation growth and EWD. Specifically, GeoDetector was used to identify which individual factors and factor interactions had stronger explanatory power for the spatial heterogeneity of NDVI–EWD coupling. Therefore, the GeoDetector results provided a basis for factor screening and interaction interpretation before the subsequent path analysis.

#### 2.3.8 Partial least squares structural equation modeling (PLS-SEM)

PLS-SEM is a variance-based structural equation modeling approach that is used to study complex path relationships among multiple latent variables including its direct and indirect effects (Hair et al., 2021). Unlike GeoDetector, which focuses on detecting spatial explanatory power and factor interactions, PLS-SEM was used to further examine the potential influence pathways among different groups of driving factors. Thus, the two methods were used in a complementary manner: GeoDetector identified which factors were important, whereas PLS-SEM helped explain how these factors influenced NDVI–EWD coupling through direct and indirect pathways. Compared with conventional covariance-based structural equation modeling, PLS-SEM imposes fewer assumptions on data distribution. It also emphasizes more on explanatory and predictive performance. Thus, it is appropriate for exploratory studies, complex model structures and relatively small sample sizes (Becker et al., 2022; Ringle et al., 2023). In this study, PLS-SEM was used to analyze the pathways of influence of latent factors such as climate, terrain, soil, and anthropogenic activities on the coupling relationship between vegetation growth and EWD during the period of 2000 to 2024. Specifically, a measurement model was first constructed to define the relationship between latent variables and their observed indicators. A structural model was established to represent the path relationship among latent variables. Model performance was evaluated using the path coefficients, significance tests, coefficient of determination (R^2^), average variance extracted (AVE), composite reliability (CR) and variance inflation factor (VIF), and the direct and indirect effects of different driving factors on the coupling coordination relationship between NDVI and EWD were identified.

## 3 Results

### 3.1 Characterization of NDVI and EWD dynamics

#### 3.1.1 Patterns of spatiotemporal changes in NDVI

**Figure 3:**
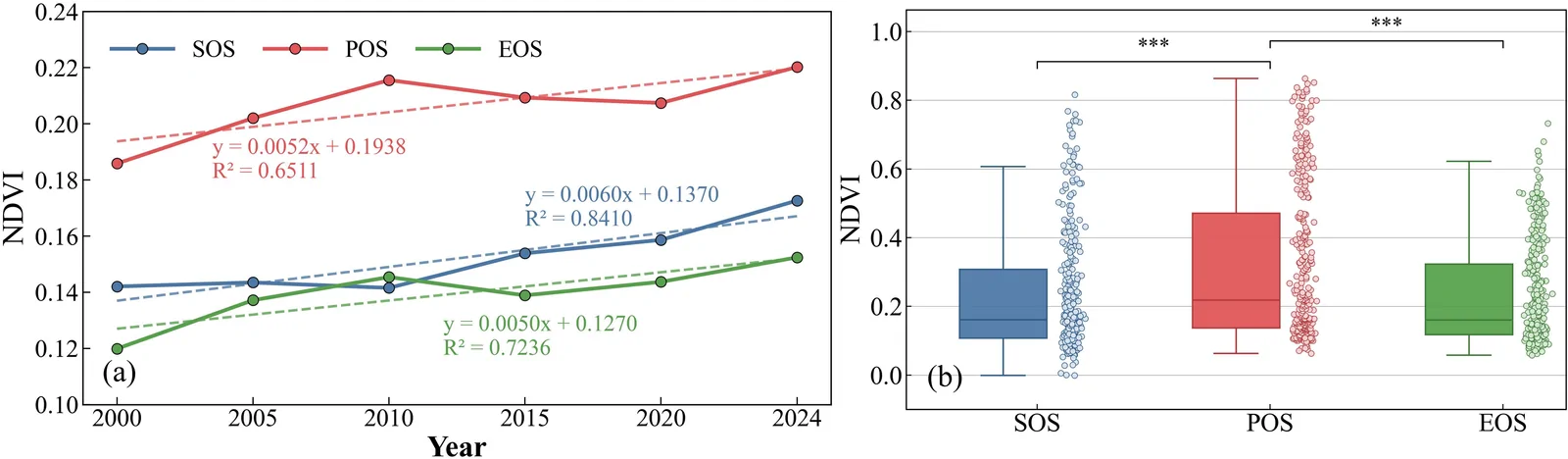
Spatiotemporal changes in mean NDVI during the SOS, POS, and EOS stages from 2000 to 2024. **(a)** Temporal trends in NDVI. **(b)** boxplots of NDVI.

The spatiotemporal distribution of NDVI in Xinjiang at different phenological stages is shown in **Figure 3**, with all the mean NDVI values at different phenological stages exhibiting fluctuating upward trends (SOS: y = 0.006x + 0.137, R^2^ = 0.841; POS: y = 0.0052x + 0.1938, R^2^ = 0.6511; EOS: y = 0.005x + 0.127, R^2^ = 0.7236), indicating the overall greening trend of the study area (**Figure 3a**). The NDVI distributions of different phenological stages were obviously different (**Figure 3b**). The NDVI value at the POS stage was the highest among the three phenological stages, with a median value of 0.2185 (Q1 = 0.1367, Q3 = 0.4711). The interquartile range at the POS stage (IQR = 0.3344) was also larger than that at the SOS stage (IQR = 0.2005) and EOS stage (IQR = 0.2056), indicating that vegetation growth was the strongest during the peak growing season, and the spatial heterogeneity was the highest.

**Figure 4:**
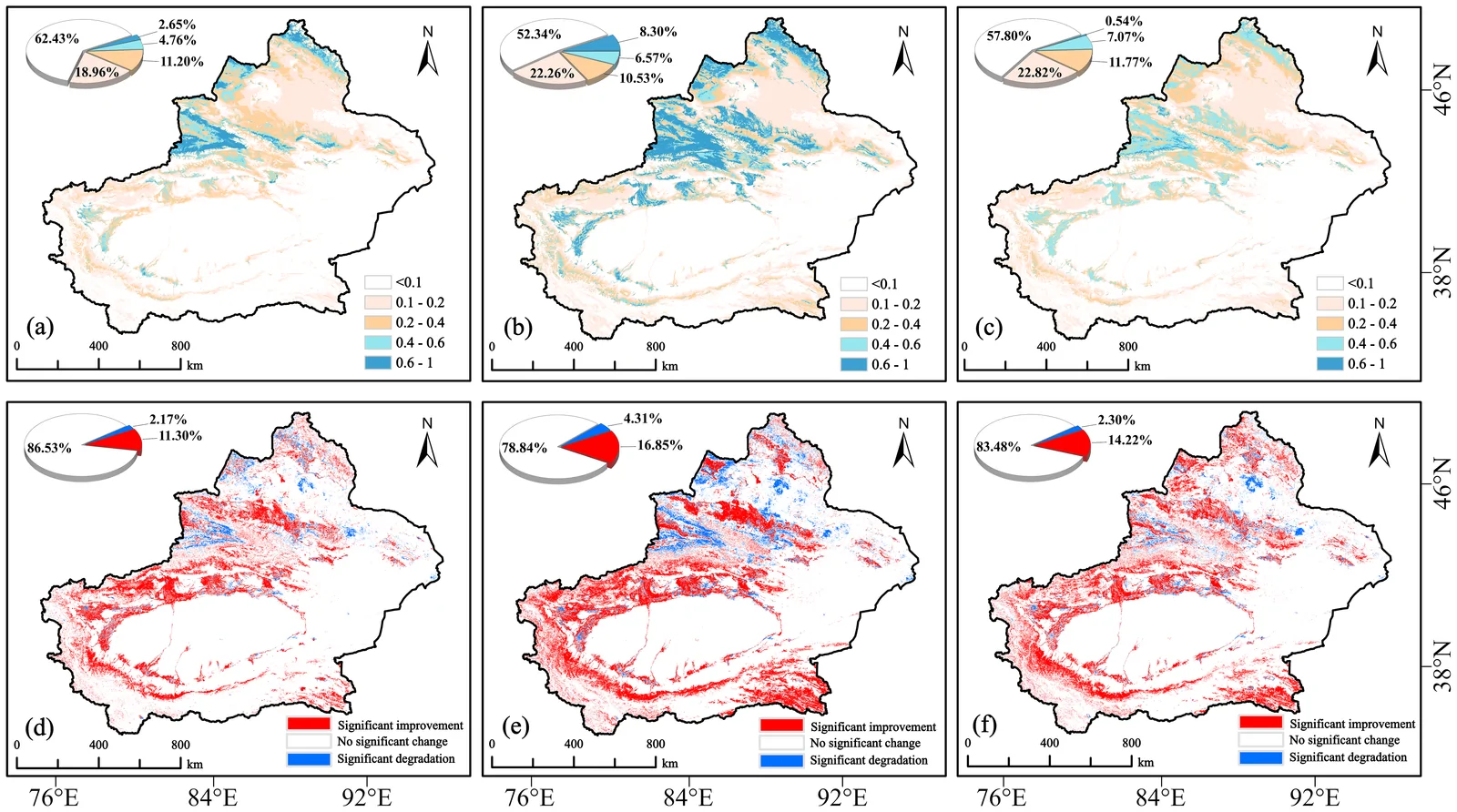
Spatial distribution and trend patterns of mean NDVI during different phenological stages from 2000 to 2024. (a–c) Spatial distribution of mean NDVI during the SOS, POS, and EOS stages. The classification criteria were as follows: I ≤ 0.1; 0.1< II ≤ 0.2; 0.2< III ≤ 0.4; 0.4< IV ≤ 0.6; 0.6< V ≤ 1. (d–f) Sen–MK trend test of NDVI during the SOS, POS, and EOS stages.

The spatial distribution of mean NDVI during 2000–2024 presented significant regional heterogeneity for the three phenological stages (**Figures 4a–c**). Across all three phenological stages, areas classified as NDVI Classes I and II were mainly distributed in desert regions, including the Tarim Basin, Junggar Basin, and Turpan–Hami Basin. These areas formed the dominant sparse-vegetation background of Xinjiang. In contrast, areas classified as NDVI classes III, IV, and V were mainly concentrated in the Tianshan Mountains, Altai Mountains, northern foothills of the Kunlun Mountains, Ili River Valley, and oasis agricultural areas. During the SOS stage (**Figure 4a**), I and II areas accounted for 62.43% and 18.96% of the total area. Low-NDVI areas were dominant, showing weak vegetation activity during the early growing season. During the POS stage (**Figure 4b**), the proportion of I areas decreased to 52.34%, whereas the proportions of IV and V areas increased to 6.57% and 8.30%. This pattern indicates that vegetation growth reached its most active stage during POS. During the EOS stage (**Figure 4c**), the proportions of I and II areas increased again to 57.79% and 22.82%. Low-NDVI areas expanded again. However, IV areas still accounted for 7.07%, suggesting that oasis croplands and some mountain grasslands maintained a certain of vegetation activity. In contrast, V areas accounted for only 0.54%, reflecting a marked contraction of high-cover vegetation.

The NDVI trend patterns also showed that stable areas dominated across all phenological stages (**Figures 4d–f**), accounting for 78.84%–86.53% of the study area. Among the areas that changed, the proportion of significantly improved areas was much higher than that of significantly degraded areas. Significantly improved areas accounted for 11.30%, 16.85%, and 14.22% during the SOS, POS, and EOS stages. In contrast, significantly degraded areas accounted for only 2.17%, 4.31%, and 2.30%. These results show that vegetation change was generally characterized by improvement. Spatially, the improvement signal was particularly evident in southern Xinjiang. A relatively continuous improvement belt was observed along the margins of the Tarim Basin. Significantly degraded areas covered a smaller extent and showed a scattered mosaic pattern. These areas were mainly found in mountain grasslands of northern Xinjiang, on the northern slope of the Tianshan Mountains, and in local areas along the southern margin of the Junger Basin.

#### 3.1.2 Patterns of spatiotemporal changes in EWD

**Figure 5:**
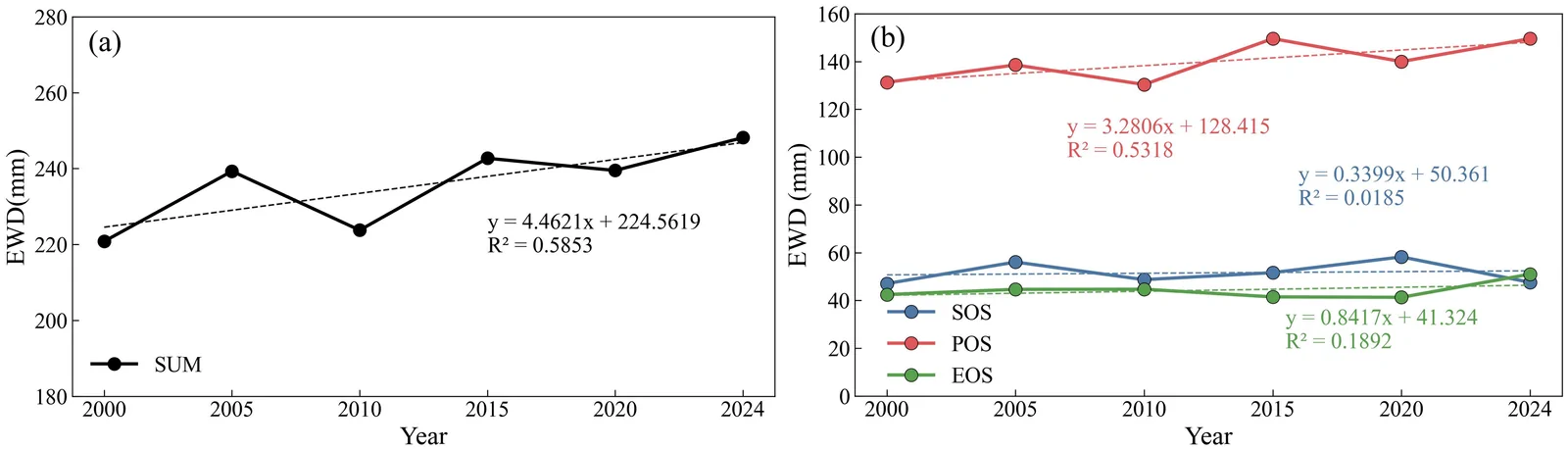
Spatiotemporal patterns of EWD during the SOS, POS, and EOS stages in Xinjiang from 2000 to 2024. **(a)** Temporal variation in growing-season EWD. **(b)** Temporal variation in EWD across different phenological stages.

The annual average EWD of various phenological stages in Xinjiang from 2000 to 2024 was 231.48~256.25 mm, with a fluctuating upward trend (**Figure 5a**). Significant differences existed in phenological stages. The multi-year average EWD values of SOS, POS and EOS stages were 51.55 mm, 139.90 mm and 44.27 mm. The EWD of the POS stage accounted for the largest proportion and was the main period of EWD in the year (**Figure 5b**). The time series results showed relatively small interannual fluctuations in EWD during the SOS and EOS stages. A more pronounced increasing trend was observed during the POS stage.

**Figure 6:**
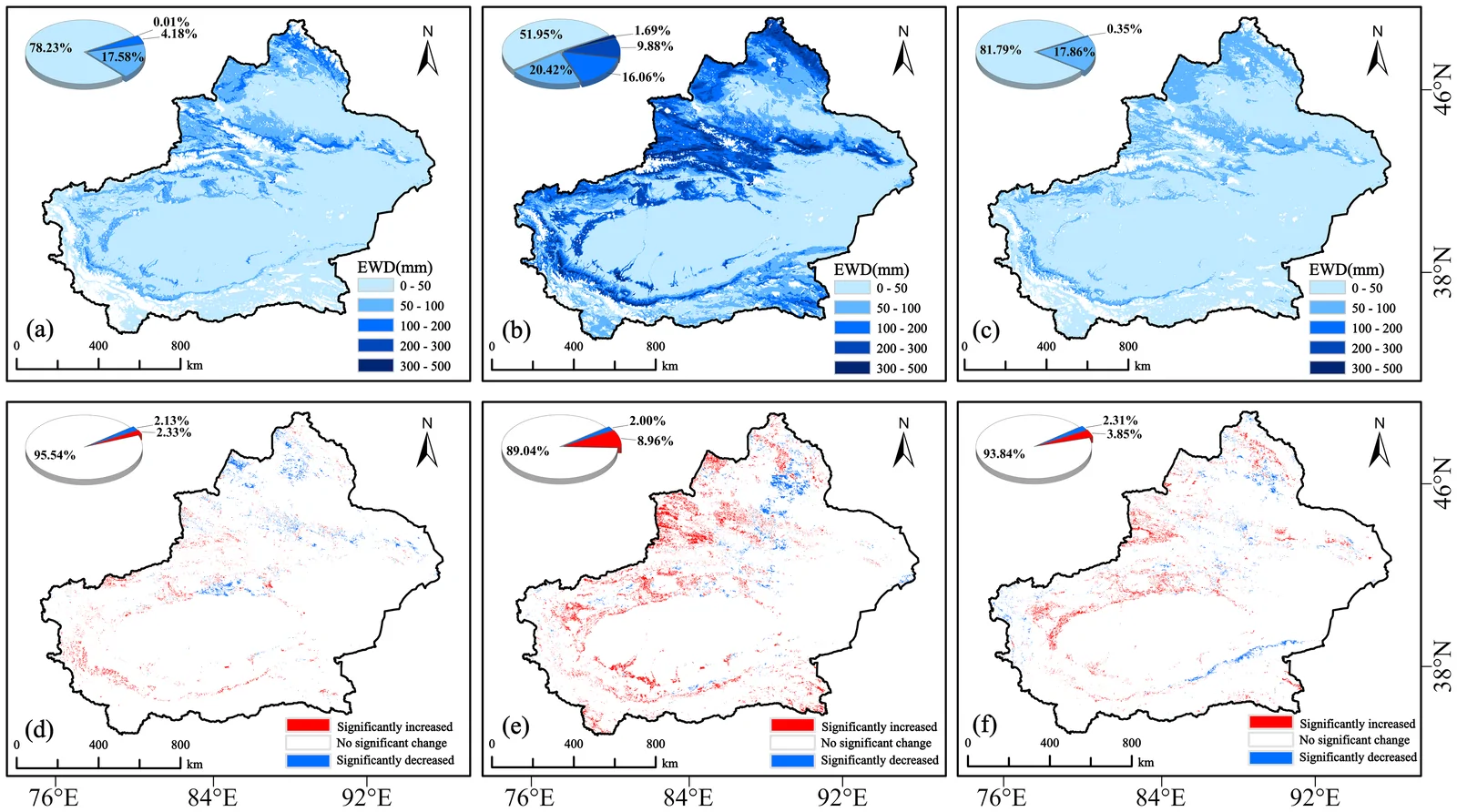
Spatial distribution and trend patterns of EWD across different phenological stages from 2000 to 2024. (a–c) Spatial distribution of mean EWD during the SOS, POS, and EOS stages. (d–f) Sen–MK trend test of EWD during the SOS, POS, and EOS stages.

**Figure 6** illustrates the spatial distribution and trend pattern of EWD in different phenological periods in Xinjiang in 2000–2024. A clear spatial gradient was observed, EWD increased from the interiors of the basins toward mountain areas, piedmont zones, and oasis regions. Areas with high EWD were mainly distributed in the mountains of northern Xinjiang, along the Tianshan Mountains, on the northern foothills of the Kunlun Mountains, and in oasis agricultural areas. In contrast, areas with limited EWD dominated the interiors of the Tarim Basin, Junggar Basin, and Turpan–Hami Basin. This pattern reflects the combined effects of hydrothermal conditions, vegetation cover, and oasis distribution on EWD in arid regions.

Across phenological stages, EWD was relatively low during the SOS stage (**Figure 6a**). Areas with EWD values of 0–50 mm accounted for 78.23% of the study area, and areas with high EWD were scattered mainly along mountain areas and piedmont transition zones. During the POS stage (**Figure 6b**), EWD increased markedly. The proportion of areas with EWD values of 0–50 mm decreased to 51.95%, whereas the proportions of areas with EWD values of 100–200 mm, 200–300 mm, and 300–500 mm increased to 16.06%, 9.88%, and 1.69%. This shift shows that vegetation water consumption increased during the peak growing season, and areas with high EWD expanded accordingly. During the EOS stage (**Figure 6c**), the proportion of areas with EWD values of 0–50 mm increased to 81.79%, whereas areas with EWD values of 100–200 mm accounted for only 0.35%. This indicates that EWD declined across most of the study area at the end of the growing season, although relatively high-water demand persisted in mountain areas and some oasis regions.

The trend analysis of EWD showed that the dominant areas with nonsignificant changes were observed in all three phenological stages during 2000 to 2024, with a proportion of 89.04%–95.54% of the study area. This indicated that the spatial structure of EWD in Xinjiang remained generally stable during the study period. EWD of the POS stage showed the most obvious increase among the three phenological stages, with the areas having a significant increase accounting for 8.96% of the study area, which was higher than the proportions during the SOS stage (2.13%) and EOS stage (2.31%). These regions were mainly distributed in patches or belts along the mountains of northern Xinjiang, Tianshan Mountains and piedmont oasis zones. In contrast, the magnitude of change during the SOS stage was relatively limited, with significantly increased and significantly decreased areas accounting for 2.13% and 2.33%. During the EOS stage, significantly increased and significantly decreased areas accounted for 2.31% and 3.85%, and were generally scattered. For the study area as a whole, EWD in Xinjiang remained largely stable during the study period. However, a clear increase was observed during the POS stage, together with localized increases and decreases in other areas. The increase in EWD during POS represented the main signal of enhanced water demand during the growing season.

### 3.2 Coupling relationship between NDVI and EWD

#### 3.2.1 Spatiotemporal evolution of the CCD

**Figure 7:**
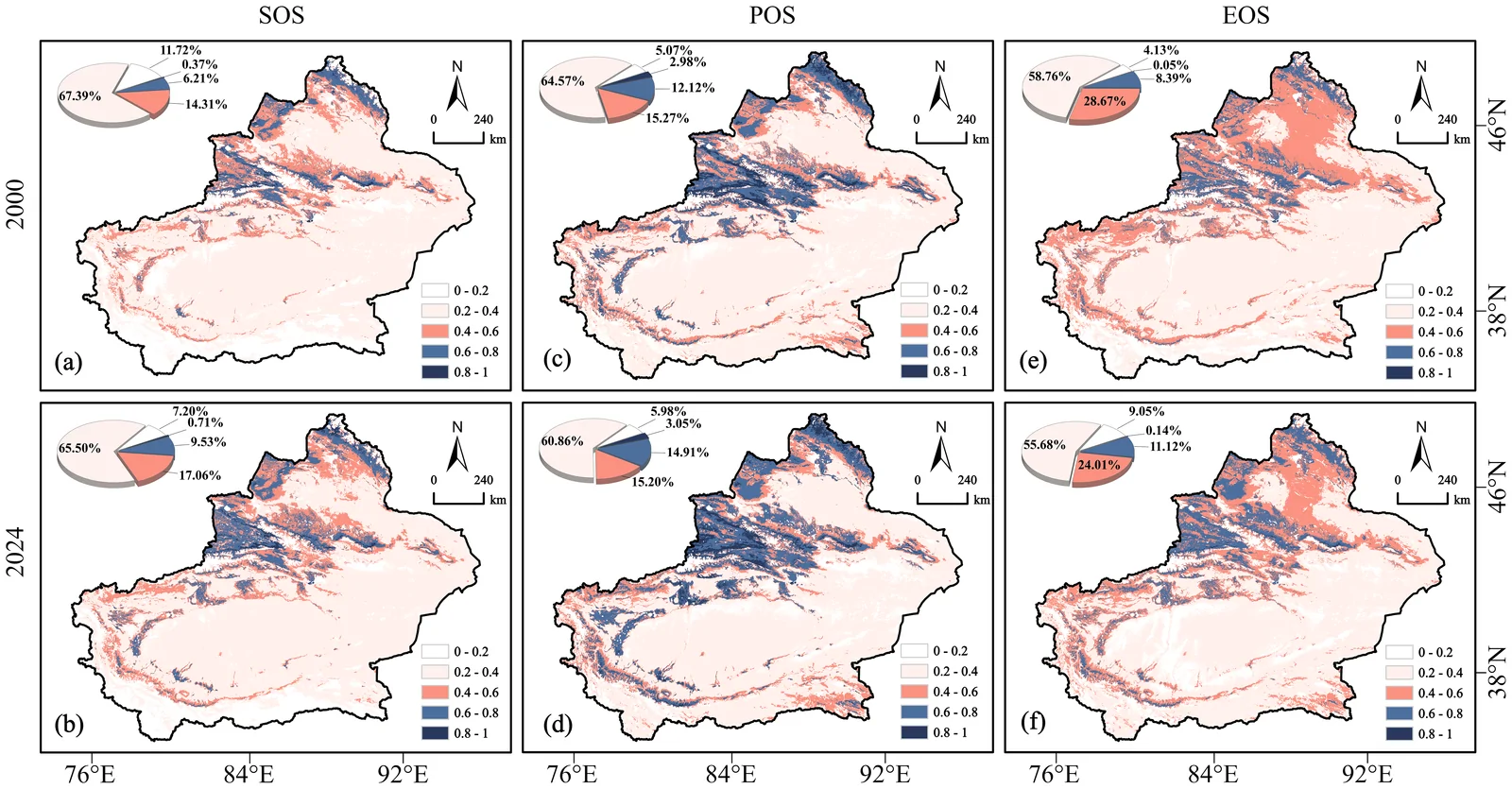
Spatiotemporal distribution of the CCD between NDVI and EWD during the **(a, b)** SOS, **(c, d)** POS, and **(e, f)** EOS stages in Xinjiang from 2000 to 2024.

The CCD model was applied to quantify the coordinated relationship between NDVI and EWD to explore the coupling coordination levels and changes in different phenological periods (**Figure 7**). At the regional scale, the CCD between NDVI and EWD was characterized by obvious spatial differentiation in Xinjiang from 2000 to 2024. The areas with higher coordination levels were mainly distributed in areas characterized by good vegetation growth conditions, such as the Altai Mountains, the Tianshan Mountains and their piedmont zones, and the Ili River Valley. In contrast, imbalanced areas were mainly concentrated in sparsely vegetated regions, such as the interior of the Tarim Basin, the interior of the Junggar Basin, and the Turpan–Hami Basin. This spatial pattern suggests that the spatial coupling between vegetation conditions and EWD in arid Xinjiang was strongly shaped by the mountain–oasis–desert landscape structure.

The changes in the proportions of areas showed that the CCD generally improved across phenological stages, but the magnitude of improvement and the patterns specific to each stage were different. During the SOS stage (**Figures 7a, b**), the proportions of serious imbalance and moderate imbalance areas decreased from 12% and 68% to 7% and 65%. In contrast, the proportions of basic coordination and moderate coordination increased from 14% and 6% to 17% and 10%, which suggested a clear improvement in coupling coordination during the early growing season. During the POS stage (**Figures 7c, d**), the proportion of moderate imbalance areas decreased from 65% to 61%, and the proportion of moderate coordination increased from 12% to 15%, indicating a steady improvement in coupling coordination during the POS. During the EOS stage (**Figures 7e, f**), the proportion of moderate imbalance areas decreased from 59% to 56%, and that of moderate coordination areas increased from 8% to 11%. However, the ratio of serious imbalance areas increased from 4 to 9% and the ratio of basic coordination areas decreased from 29 to 24%. These results indicate that the EOS stage did not show a unidirectional improvement of coupling coordination but localized improvement and localized degradation occurred simultaneously.

**Figure 8:**
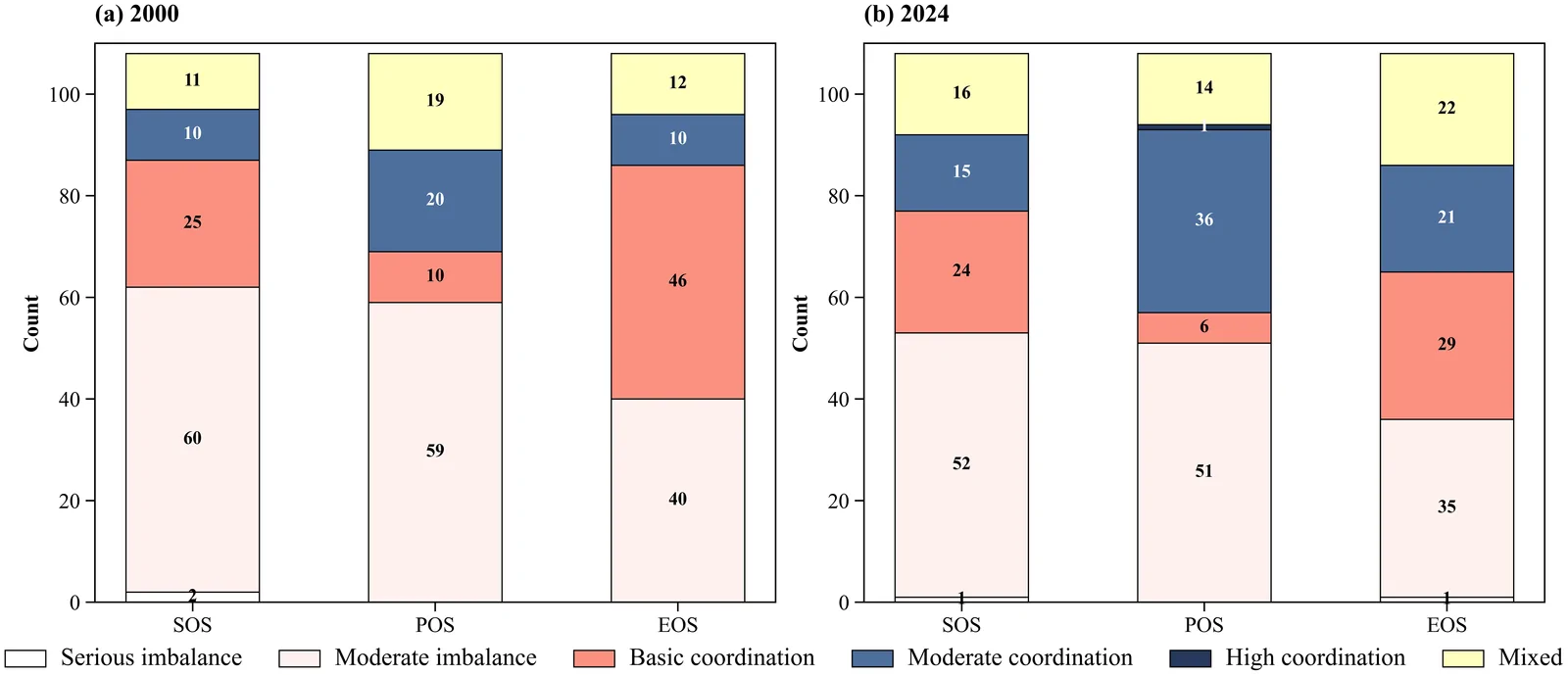
County level distribution of CCD classes during the SOS, POS, and EOS stages in **(a)** 2000 and **(b)** 2024.

The county level results showed a contraction of imbalanced areas during the three phenological stages (**Figure 8**). From the SOS, POS and EOS stages, the number of counties classified as moderate imbalance decreased from 60, 59 and 40 to 52, 51 and 35. Conversely, the number of counties classified as moderate coordination increased from 10, 20 and 10 to 15, 36 and 21. These changes further indicate an overall improvement in the coupling coordination status between NDVI and EWD in Xinjiang during the study period. The improvement was most significant during the POS stage.

**Figure 9:**
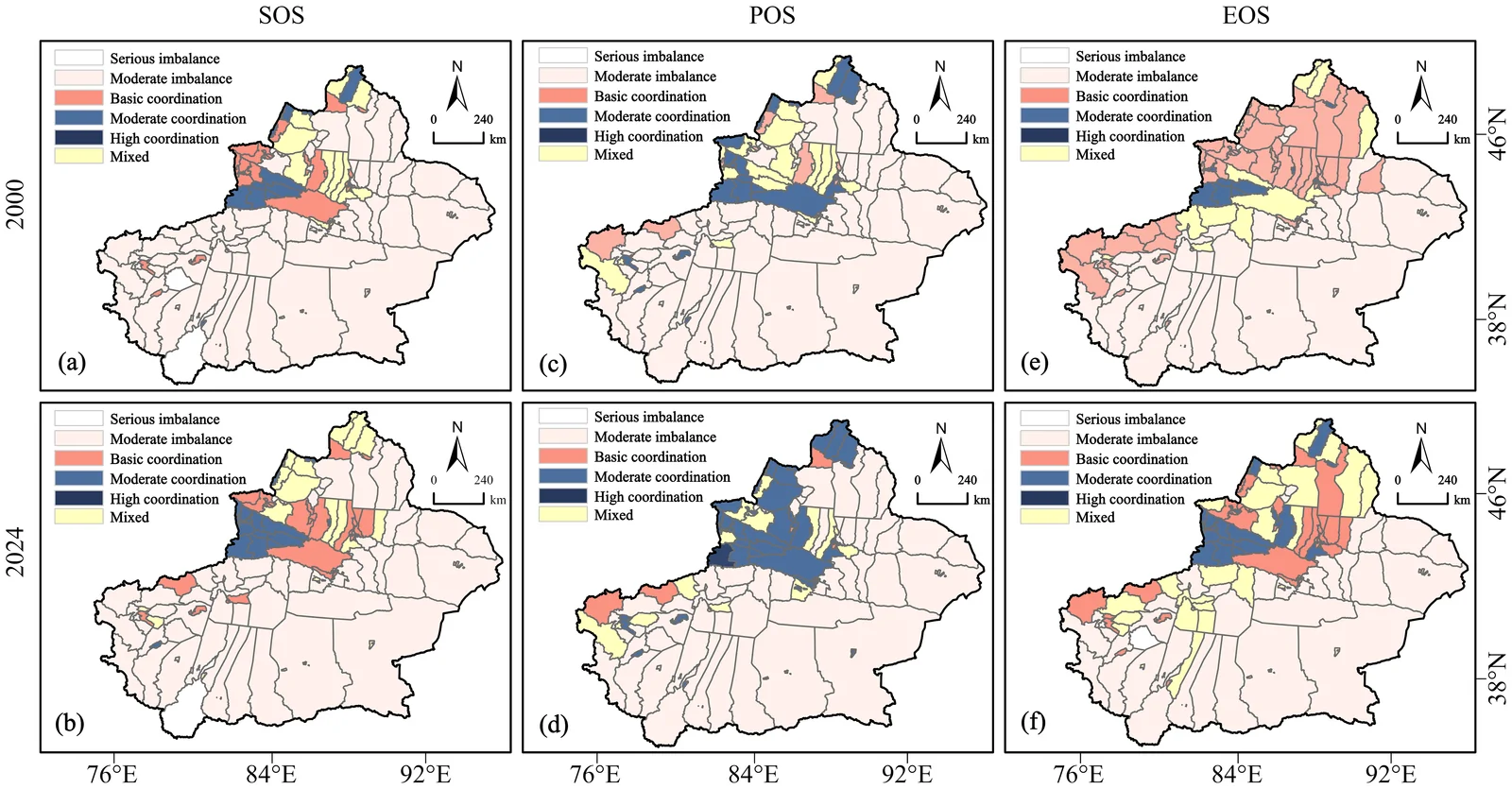
County-level spatiotemporal distribution of CCD in 2000 and 2024 during the **(a, b)** SOS, **(c, d)** POS, and **(e, f)** EOS stages.

County level dominant type transitions showed clear differences among phenological stages. During the SOS stage (**Figures 9a, b**), some counties shifted from moderate imbalance to mixed types, including Jimar County, Yuepuhu County, and Tiemenguan City. In the northwestern Ili River Valley, several counties and county level cities shifted from basic coordination to moderate coordination. These changes suggest that the improvement in coupling coordination during the SOS stage was mainly reflected in local upgrades of county-level coordination classes, whereas the broader spatial pattern remained under gradual adjustment. During the POS stage (**Figures 9c, d**), county level type transitions were the most pronounced. The number of moderate imbalance counties decreased markedly, whereas basic coordination and moderate coordination counties increased. Dushanzi District shifted from moderate imbalance to basic coordination. Kashgar City, Kuytun City, and Tiemenguan City shifted from moderate imbalance to moderate coordination. Kokdala City, Shawan City, and Shuanghe City shifted from basic coordination to moderate coordination. These transitions indicate that improvements in county level coupling coordination were more concentrated during the POS. During the EOS stage (**Figures 9e, f**), improvements in coordination levels were mainly observed in the Ili River Valley and parts of northern Xinjiang. For example, Burqin County, Nilka County, and Urumqi County shifted from mixed types to moderate coordination. Some counties and county level cities in the Ili River Valley also shifted from basic coordination to moderate coordination. In this stage, spatial differences were more obvious. This pattern shows that the coupling coordination at the end of the growing season was more influenced by the regional differences in vegetation senescence and water availability.

#### 3.2.2 Spatiotemporal differentiation of quadrant coupling types

**Figure 10:**
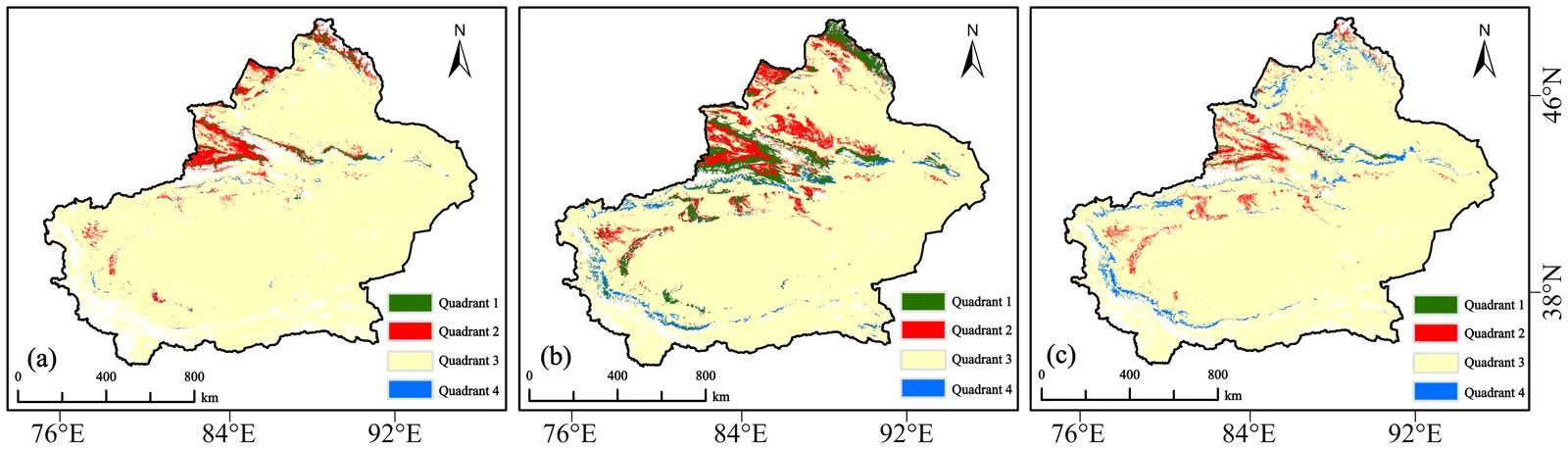
Spatiotemporal patterns of NDVI–EWD coupling types in Xinjiang during the **(a)** SOS, **(b)** POS, and **(c)** EOS stages from 2000 to 2024. EWD is shown on the horizontal axis, and NDVI is shown on the vertical axis.

Using the quadrant analysis (**Figure 10**), we identified the coupling types between NDVI and EWD. The HH type in the first quadrant (high NDVI and high EWD) was mainly observed in the POS stage. The HH type was mainly observed in the Ili River Valley, the middle and high elevation zones of the Altai Mountains, and the mountainous regions along the western and northern slopes of the Tianshan Mountains. The HL type in the second quadrant (high NDVI and low EWD) was observed in all three phenological stages. It was mainly distributed on the southern slope of the Altai Mountains, the northern slope of the Tianshan Mountains, the margins of the Ili River Valley, and some oasis areas along basin edges. These areas had relatively high vegetation cover. The third quadrant, characterized by LL (low NDVI and low EWD), was mainly distributed in the interior of the Taklimakan Desert, the Gurbantunggut Desert, and the arid basins of eastern Xinjiang. This type represented the typical desert background in Xinjiang. The fourth quadrant, characterized by LH (low NDVI and high EWD), was mainly distributed along the northern and western margins of the Tarim Basin, the Tarim River, the piedmont zones of the southern Tianshan Mountains, and local margins of the Turpan–Hami Basin. These areas showed a mismatch between vegetation restoration and water demand. Taken together, the quadrant distribution further revealed spatial differences in vegetation and water relationships across Xinjiang. The HH type were more likely to occur in mountain and oasis regions, whereas the LL type persisted in basin interiors. In some desert–oasis transition zones, relatively high water-demand pressure may indicate potential water-constraint risks.

**Figure 11:**
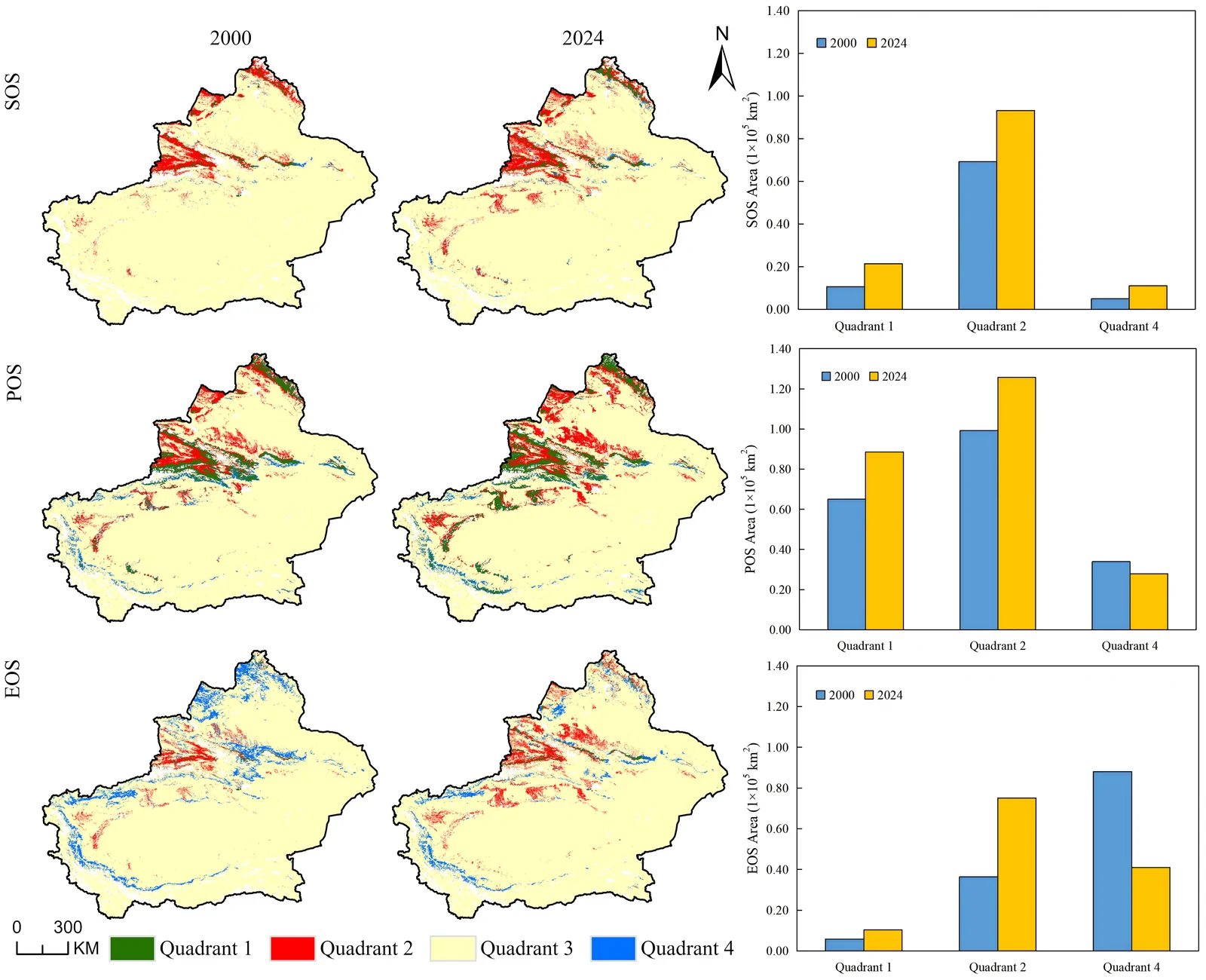
Spatiotemporal distribution of NDVI–EWD coupling types during the SOS, POS, and EOS stages in Xinjiang in 2000 and 2024.

The LL coupling type was the dominant coupling type of NDVI and EWD in Xinjiang from 2000 to 2024 in phenological stages, but the area of the LL coupling type generally reduced (**Figure 11**). The area of LL decreased from 1.459 × 10^6^ km² to 1.345 × 10^6^ km² at the SOS stage, while the areas of the HH, HL, and LH coupling types all increased. During the POS stage, the LL area further decreased to 1.266 × 10^6^ km². In contrast, the areas of HH and HL increased to 8.849×10^4^ km² and 1.257×10^5^ km², whereas the LH area decreased to 2.789×10^4^ km². This pattern shows a shift toward coupling types associated with higher NDVI, together with a contraction of mismatched LH areas. During the EOS stage, the LL area decreased to 1.371×10^6^ km², while HH and HL continued to expand. The LH area decreased markedly from 8.798×10^4^ km² to 4.093×10^4^ km², indicating an improvement in the vegetation–water demand relationship in some areas at the end of the growing season. Thus, NDVI–EWD coupling types in Xinjiang were characterized by LL dominance, expansion of HH and HL, and contraction of LH. The most active type conversion occurred during the POS stage, when spatial differentiation among coupling types was most pronounced.

**Figure 12:**
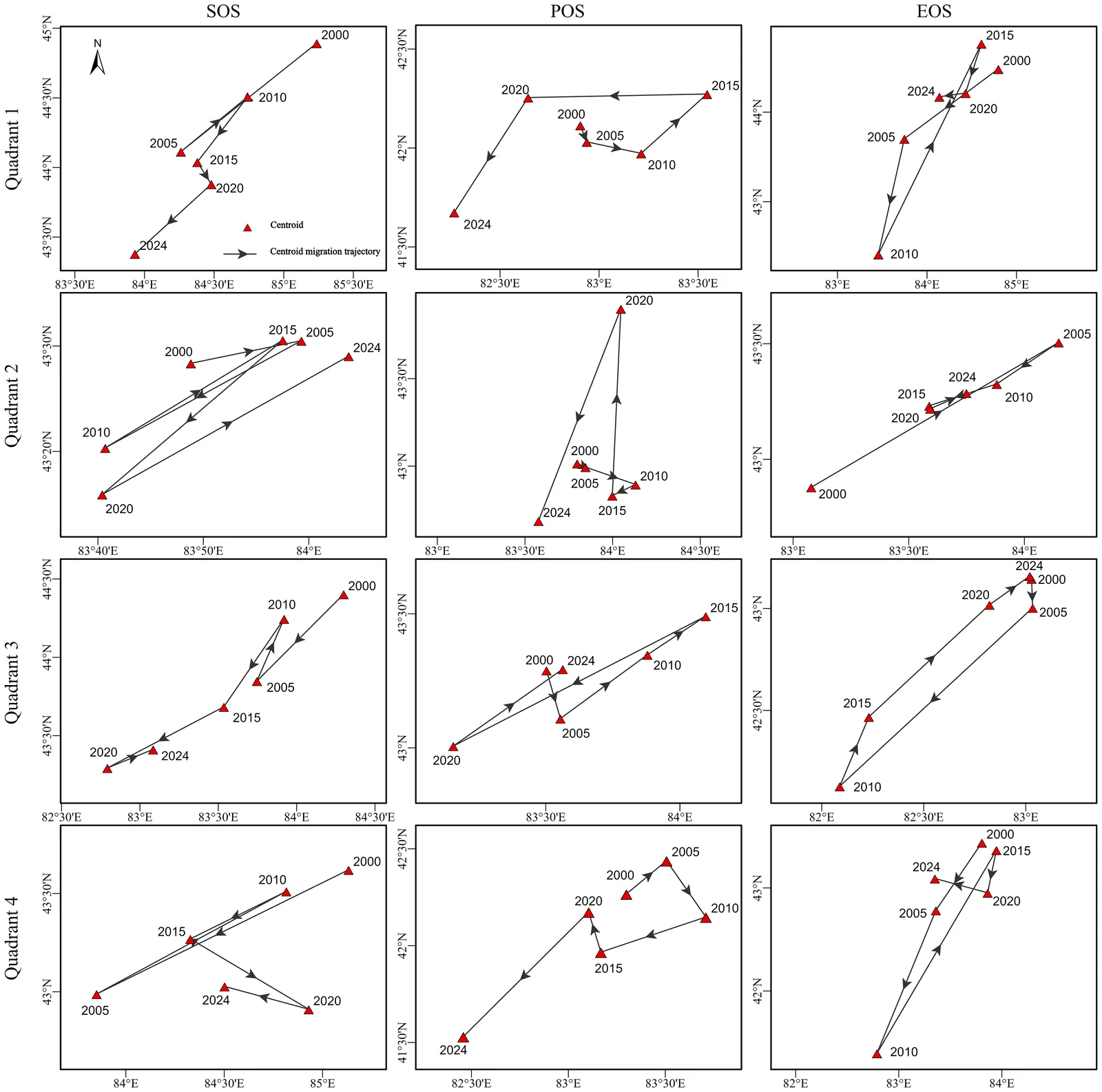
Spatial centroid migration paths of NDVI–EWD coupling types from 2000 to 2024.

The results of centroid migration further revealed the spatial reorganization of NDVI–EWD coupling types (**Figure 12**). During the period from 2000 to 2024, some types of quadrant centroids moved southwest, indicating that the partial movement of these coupling types was toward southern Xinjiang during the study period. However, northward adjustments or return-like movements were also observed for some quadrant types. Notably, although the LL centroid showed stage-specific fluctuations across the three phenological stages, its net positional change was relatively limited. This pattern indicates that the LL type, as the dominant background type in the desert regions of Xinjiang, had strong spatial stability.

The transition matrices of the four quadrant types from 2000 to 2024 showed the dominance of LL to HL transitions at all phenological stages, with 53.95%, 35.86%, and 31.87% of the total transformed area during the SOS, POS, and EOS stages. During the SOS stage (**Table 3**), transitions from HL to LL and from HL to HH were also evident, with areas of 1.013×10^4^ km² and 9.709×10^3^ km². During the POS stage (**Table 4**), the transition pathways became more diverse. The transition areas from HL to HH, HL to LL, LL to HH, LL to LH and LH to LL were 2.082×10^4^ km², 1.331×10^4^ km², 1.116×10^4^ km², 1.114×10^4^ km² and 1.014×10^4^ km². This phenomenon showed that the bidirectional conversion and multi-pathway differentiation between quadrant types were most significant during the POS stage, which means that the POS stage was the most active period of coupling type reorganization. During the EOS stage (**Table 5**), type evolution was generally characterized by a return toward the dominant type. The transition from LH to LL was the most prominent, with an area of 5.283×10^4^ km². In addition, the transition areas from LL to HL, LL to LH, and HL to LL were 45,564.97 km², 1.623×10^4^ km², and 1.107×10^4^ km². These results suggest that some LH areas reverted to LL gradually after the growing season, while a significant fraction of LL areas were still converted to HL.

**Table 3:** Transfer matrix of quadrant-based coupling types during the SOS stage from 2000 to 2024.

2000—2024	HH	HL	LL	LH
HH	7707.61	1669.35	372.21	514.60
	(—)	(2.11%)	(0.47%)	(0.65%)
HL	9709.65	45985.55	10313.85	1001.76
	(12.26%)	(—)	(13.02%)	(1.27%)
LL	2725.48	42722.58	1389087.38	7293.74
	(3.44%)	(53.95%)	(—)	(9.21%)
LH	574.81	378.27	1910.32	1540.94
	(0.73%)	(0.48%)	(2.41%)	(—)

Values in parentheses indicate the percentage of each transition relative to the total area of changed pixels. Stable classes, such as HH to HH, were excluded from the percentage calculation; Rows represent coupling types in 2000, and columns represent coupling types in 2024. The values indicate the net transition area between the two years.

**Table 4:** Transfer matrix of quadrant-based coupling types during the POS stage from 2000 to 2024.

2000-2024	HH	HL	LL	LH
HH	49231.80	7738.65	3467.69	4611.31
	(—)	(5.21%)	(2.33%)	(3.10%)
HL	20824.32	61432.40	13316.66	1334.22
	(14.02%)	(—)	(8.96%)	(0.90%)
LL	11160.61	53282.73	1302705.84	11141.71
	(7.51%)	(35.86%)	(—)	(7.50%)
LH	9746.04	1813.37	10144.39	10773.04
	(6.56%)	(1.22%)	(6.83%)	(—)

**Table 5:** Transfer matrix of quadrant-based coupling types during the EOS stage from 2000 to 2024.

2000-2024	HH	HL	LL	LH
HH	2216.59	1847.67	986.40	435.98
	(—)	(1.29%)	(0.69%)	(0.30%)
HL	2625.90	20396.12	11068.17	293.88
	(1.84%)	(—)	(7.74%)	(0.21%)
LL	2680.96	45564.97	1351160.12	16231.81
	(1.88%)	(31.87%)	(—)	(11.35%)
LH	2530.44	5877.82	52826.88	24667.40
	(1.77%)	(4.11%)	(36.95%)	(—)

### 3.3 Driving factors of NDVI–EWD coupling

#### 3.3.1 Factor detection results from GeoDetecto

**Figure 13:**
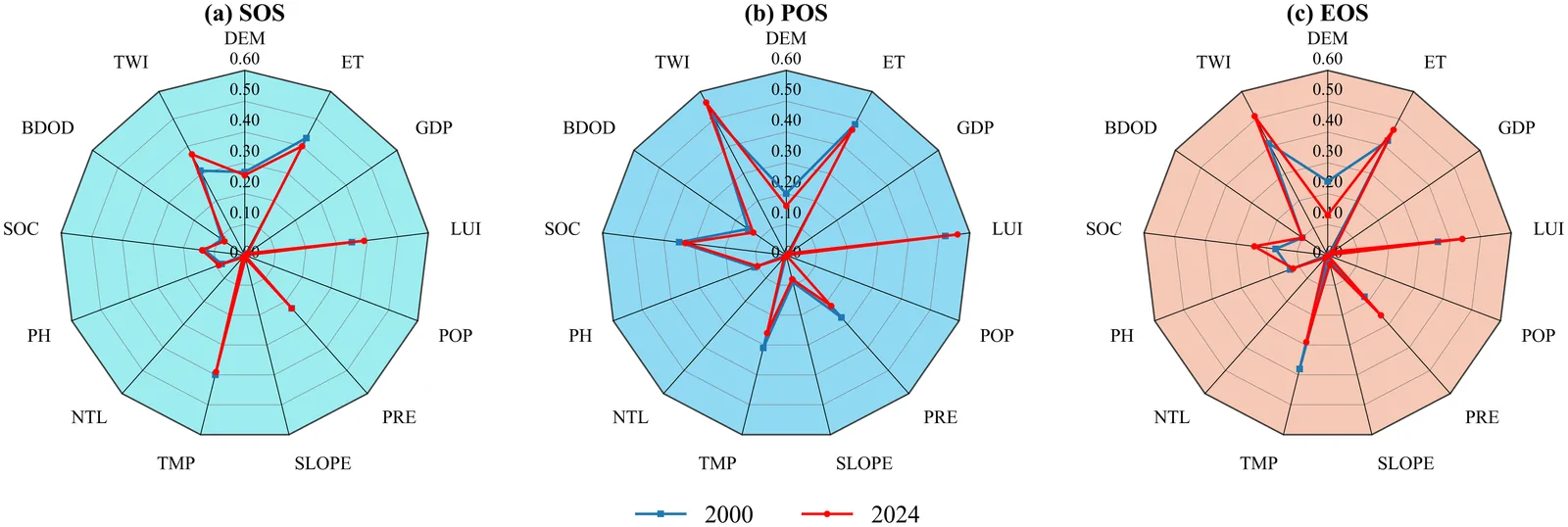
Factor detector results for NDVI–EWD coupling in Xinjiang during the **(a)** SOS, **(b)** POS, and **(c)** EOS stages from 2000 to 2024. (P<0.05).

The results of factor detection indicated that the spatial differentiation of NDVI-EWD coupling in Xinjiang from 2000 to 2024 had obvious phenological differences (**Figure 13**). ET, LUI, TMP, TWI, and SOC had relatively high q values, followed by DEM and PRE. In contrast, GDP, POP, NTL, pH, BDOD, and SLOPE generally showed lower explanatory power. These results indicate that the NDVI–EWD coupling pattern was jointly shaped by evapotranspiration processes, land-use patterns, hydrothermal conditions, and topography-related moisture conditions. Among these factors, LUI had high explanatory power in all phenological stages, indicating that land use pattern was an important variable in linking the effects of natural conditions and human activity.

For SOS, the main driving factors across the phenological stages were ET, TMP, LUI and TWI (**Figure 13a**). The q values of LUI and TWI increased in 2024 compared to 2000, and ET and TMP were still at relatively high levels. During the POS stage (**Figure 13b**), the explanatory power of most factors was stronger. LUI, TWI, ET, and SOC were particularly important. This suggests that the coupling relationship during the POS was more sensitive to land-use, topographic wetness, evapotranspiration processes, and soil background conditions. During the EOS stage (**Figure 13c**), ET, LUI, and TWI still showed high explanatory power. The effects of PRE and SOC increased, whereas the effect of TMP weakened. This pattern suggests that the coupling relationship at the end of the growing season gradually shifted from thermal control toward the combined regulation of moisture conditions, soil environment, and land surface characteristics. Overall, the driving mechanisms of NDVI–EWD coupling in Xinjiang showed clear phenological differences. Natural factors remained dominant, while human activities mainly affected the regional coupling pattern indirectly through land-use change.

#### 3.3.2 Interaction detection results from GeoDetector

**Figure 14:**
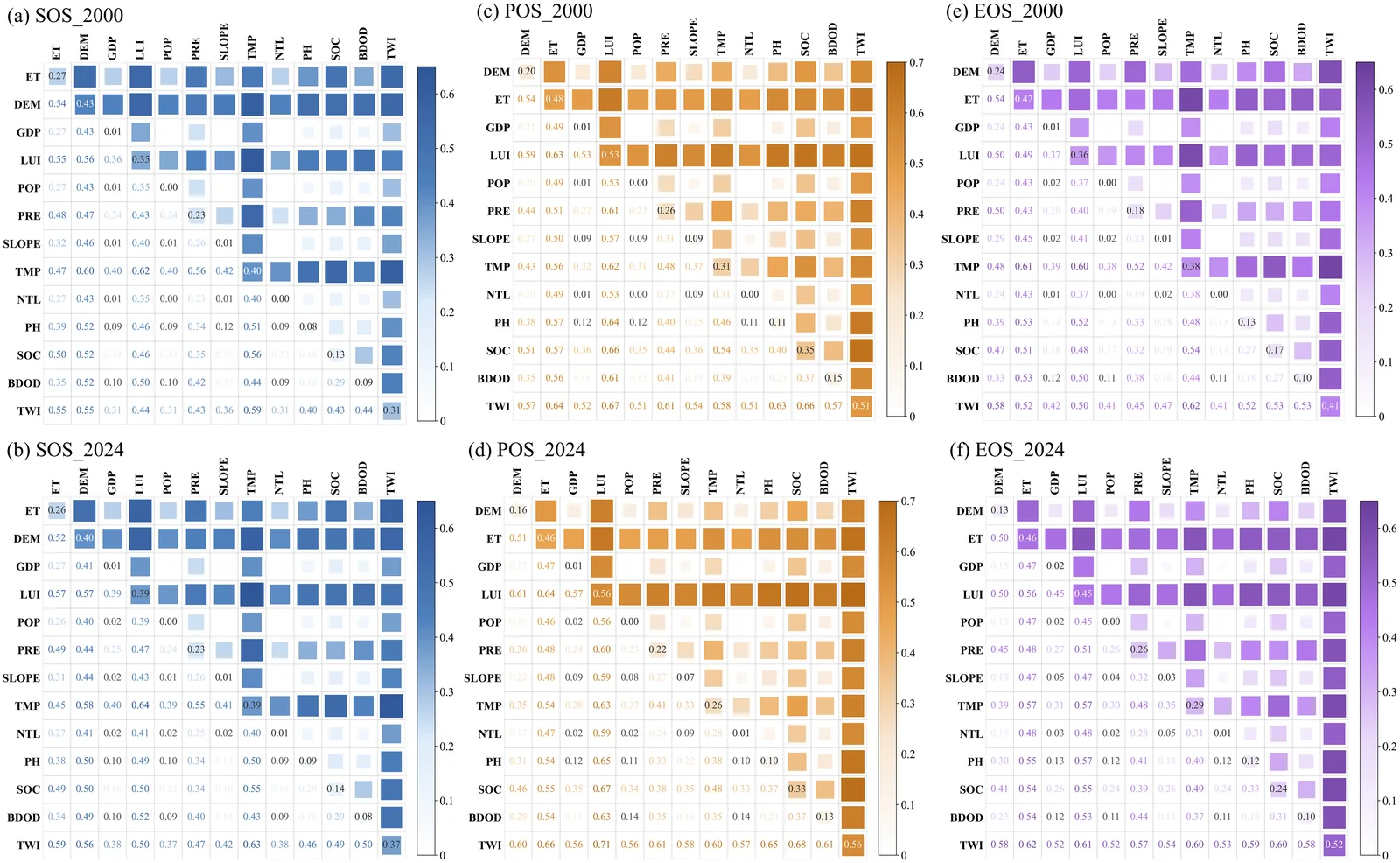
Interaction detector results for NDVI–EWD coupling in Xinjiang during the **(a, b)** SOS, **(c, d)** POS, and **(e, f)** EOS stages from 2000 to 2024. (P<0.05).

The interaction detection results showed that the spatial differentiation of NDVI–EWD coupling was not controlled by a single factor. Instead, it was shaped by the combined effects of multiple factors (**Figure 14**). For most factor pairs, the q values after interaction were higher than the explanatory power of the corresponding individual factors. This indicates that enhancement effects were common among the driving factors. High q interactions were mainly observed among LUI, TWI, ET, TMP, and soil related factors. These results suggest that the spatial heterogeneity of NDVI–EWD coupling was largely associated with the combined effects of land-use patterns, topography related moisture conditions, hydrothermal processes, and soil background conditions.

In the SOS stage (**Figures 14a, b**), the strongest interactions were mainly observed for LUI∩TMP, TWI∩TMP, DEM∩TMP, and ET∩LUI, with the highest q values observed for LUI∩TMP of 0.62 in 2000 and of 0.64 in 2024. This indicates that the coupling relationship during the early green up stage was jointly influenced by thermal conditions, evapotranspiration processes, topographic background, and land-use patterns. During the POS stage (**Figures 14c, d**), interaction effects were generally stronger. The q values of LUI∩TWI reached 0.67 in 2000 and 0.71 in 2024. In addition, SOC∩LUI, SOC∩TWI, ET∩TWI, and pH∩LUI also remained at relatively high levels. These results suggest that NDVI–EWD coupling during the peak growing season was more sensitive to land-use, topographic wetness, evapotranspiration processes, and soil conditions. Together, these patterns indicate that the POS stage was under the strongest multi-factor control. During the EOS stage (**Figures 14e, f**), the interaction q values were lower than those during the POS stage. However, ET∩TWI, LUI∩TWI, SOC∩TWI, and TMP∩TWI still showed strong explanatory power. This suggests that the coupling relationship at the end of the growing season was more strongly regulated by topographic wetness, moisture conditions, and soil background conditions.

#### 3.3.3 PLS-SEM model analysis results

**Figure 15:**
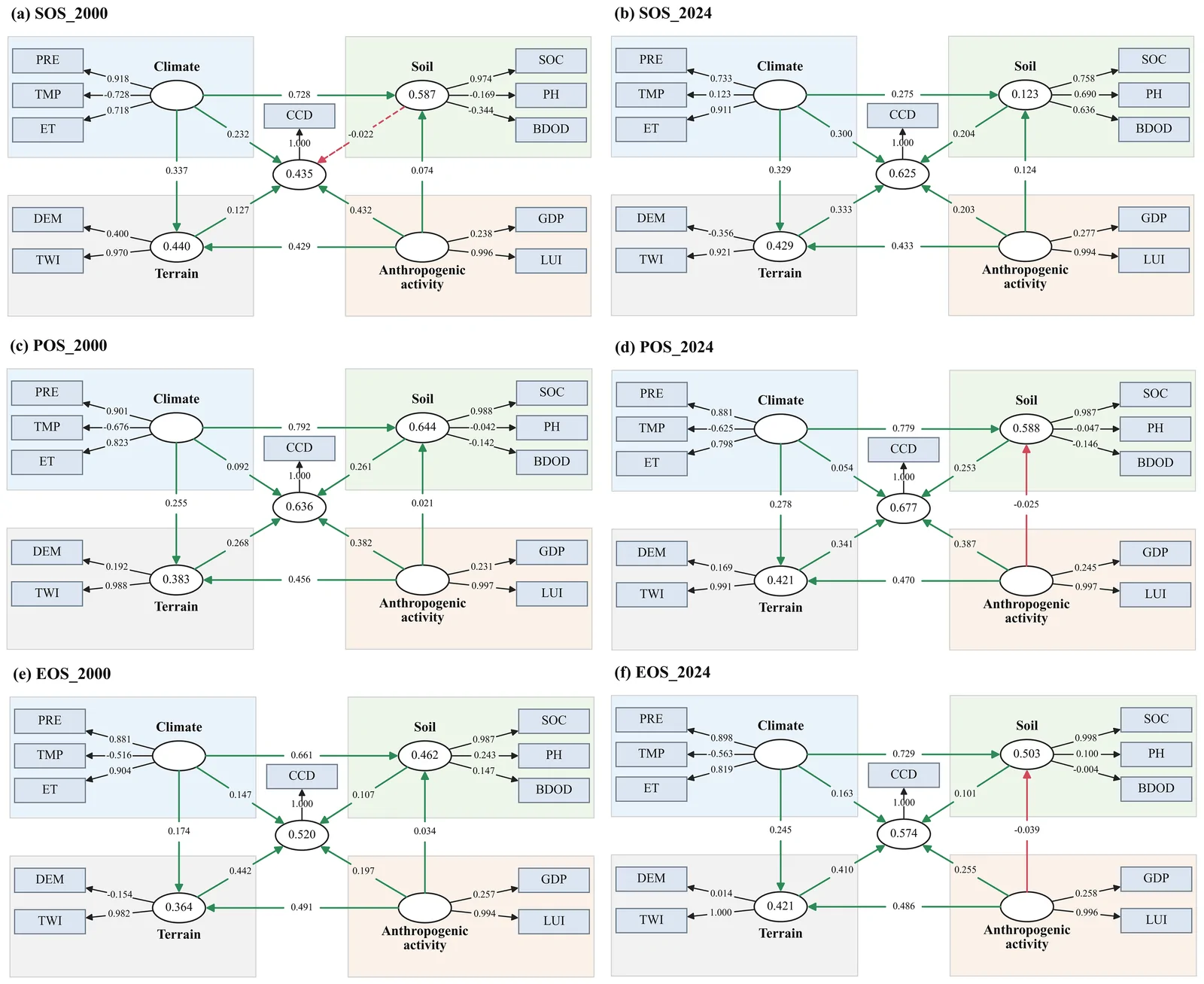
PLS-SEM path models for NDVI–EWD coupling in Xinjiang during the **(a, b)** SOS, **(c, d)** POS, and **(e, f)** EOS stages from 2000 to 2024. circles indicate latent variables, rectangles indicate observed variables, arrows represent path relationships among variables, red and green arrows denote negative and positive effects, respectively, and solid and dashed lines indicate significant paths (p< 0.001) and nonsignificant paths, respectively.

The PLS-SEM results showed that climate, terrain, soil conditions, and human activities explained the phenological differences in the CCD between NDVI and EWD in Xinjiang reasonably well (**Figure 15**). Across the models, the explained variance of CCD ranged from 0.435 to 0.677. For all three phenological stages, the values in 2024 were higher than that in 2000, which implies that the model better explained the relationship between NDVI and EWD coupling over the study period. For the three phenological stages, the highest explained variance was for the POS stage, with values of 0.636 in 2000 and 0.677 in 2024. This result indicates that the combined driving mechanisms of NDVI–EWD coupling were most clearly expressed in the peak growing season.

The structural paths showed that climate had a clear positive effect on soil conditions across all stages, with path coefficients ranging from 0.275 to 0.792. The strongest effect was observed during the POS stage. This indicates that hydrothermal conditions provided an important background for shaping the soil environment and, in turn, regulating the NDVI–EWD relationship. The direct effects of the latent variables on CCD differed markedly among phenological stages. During the SOS stage (**Figures 15a, b**), human activities had a strong direct effect on CCD in 2000. By 2024, the effects of climate, terrain, and soil conditions had increased simultaneously, and the soil path shifted from a weak negative effect to a positive effect. During the POS stage (**Figures 15c, d**), the direct effect of climate on CCD was weak, but its effect on soil conditions was the strongest. Meanwhile, terrain and human activities maintained relatively strong positive effects on CCD. This indicates that the coupling relationship in the peak growing season was affected by both the indirect climatic effects and direct effects of terrain and human activities. During the EOS stage (**Figures 15e, f**), terrain had the strongest direct effect on CCD, with path coefficients of 0.442 in 2000 and 0.410 in 2024. Human activities showed the second strongest effect, whereas the direct effects of climate and soil conditions were relatively weak. These results suggest that the coupling relationship at the end of the growing season was more strongly affected by terrain patterns and terrain controlled water redistribution.

The measurement model showed relatively consistent contributions of the observed indicators to each latent variable. Climate was mainly represented by PRE and ET. TMP showed negative loadings in most stages, suggesting that, in the arid context of Xinjiang, higher temperature may have been associated more with increased evaporative demand and intensified water stress than with a direct promotion of vegetation growth. Terrain was mainly represented by TWI, whereas the contribution of DEM was relatively weak. This indicates that the terrain effect was expressed more through water accumulation and redistribution processes than through elevation differences alone. SOC showed the highest loading for the Soil latent variable, whereas pH and BDOD had smaller contributions and showed clearer stage-specific differences. This suggests that SOC was a better representation of the soil background. Anthropogenic activity was mainly represented by LUI, whereas the loading of GDP remained low across stages. This indicates that the effect of human activities on NDVI–EWD coupling was expressed primarily through land-use patterns and land-surface use intensity, while the direct effect of differences in economic scale was relatively limited.

Taken together, the PLS-SEM results were consistent with the factor detection and interaction detection results. ET, TWI, LUI, and SOC made strong contributions in single-factor detection and also showed important effects in multi-factor interactions and structural paths. This indicates that NDVI–EWD coupling in Xinjiang was driven by a clear multi-factor mechanism. Climate conditions provided the hydrothermal background for vegetation growth and EWD. Patterns of terrain affected the accumulation and spatial redistribution of water. Soil conditions affected the basic environment of vegetation growth. Human activities mainly regulated the coupling process through land-use intensity. The dominant pathways varied among phenological stages. Natural regulation became stronger during the SOS stage, multi-factor control was most pronounced during the POS stage, and terrain-related water redistribution became more important during the EOS stage.

## 4 Discussion

### 4.1 Applicability of the EWD estimation method and reliability of the results

The reliability of NDVI–EWD coupling analysis is directly determined by the estimation method of EWD. At present, the main methods for estimating vegetation EWD mainly include the area quota method, biomass-based method, water balance method, phreatic evaporation method and remote sensing-based evapotranspiration method (Ling et al., 2014; Chi et al., 2018; Zhao et al., 2019; Fu et al., 2021). However, for large arid regions such as Xinjiang with strong landscape heterogeneity and relatively limited long-term observational records, these methods are often difficult to simultaneously meet the requirements of regional-scale assessment, long-term analysis and spatial continuity. In contrast, the FAO Penman–Monteith method-based approaches together with the information of evapotranspiration, soil moisture limitation and vegetation coefficients have been widely used to assess EWD, monitor water-stress, and analyse ecological water supplementation in arid regions (Allen et al., 1998; Hao et al., 2023; Wang et al., 2023b).

The mean annual growing season EWD of Xinjiang was 231.48–256.25 mm during the period of 2000–2024, and all phenological stages showed significant variances in this study. The EWD of the POS phase accounted for 59.35% of the entire growing season. This pattern is consistent with the general dynamics of vegetation phenology. The maximum leaf area index, canopy cover, transpiration intensity and the highest concentration of EWD and evapotranspiration are normally characteristic of the peak growing season (Ge et al., 2021; Liu et al., 2021). Studies on EWD of natural vegetation in the Tarim River Basin also indicate the peak vegetation water demand usually occurs in the middle of the growing season and increases with vegetation cover significantly (Hao et al., 2023; Huang et al., 2024). Therefore, the EWD pattern dominated by the POS is ecologically valid in our study.

Spatially, areas with high EWD in this study were mainly concentrated in the Tianshan Mountains, Altai Mountains, Ili River Valley, northern foothills of the Kunlun Mountains, and oasis agricultural areas. In contrast, areas where EWD remained relatively limited were mainly distributed in the interiors of the Tarim Basin, Junggar Basin, and Turpan–Hami Basin. The spatial pattern of EWD in Xinjiang showed a clear mountain-oasis-desert pattern, which was highly related to vegetation, hydrothermal conditions, and ecosystem types in Xinjiang (Yao et al., 2022). The high-EWD areas in Xinjiang were mainly distributed in mountain and valley areas including the Ili River Valley, Tianshan Mountains, Altai Mountains, and Kunlun Mountains, while the interior of desert basins has long been characterized by low vegetation coverage (Zhang et al., 2024b). Water resources for the piedmont oasis and valley ecosystems are mainly from precipitation, snowmelt, and glacier meltwater from mountainous areas (Zhang et al., 2023). Therefore, the higher EWD areas we identified were closely associated with higher vegetation greenness and more stable ecological water supplies. This spatial consistency indicates that the EWD estimates well captured the regional differentiation of vegetation water demand in Xinjiang.

### 4.2 Phenological differences in NDVI–EWD coupling evolution and associated water constraints

In this study, stage-specific changes of the CCD between NDVI and EWD were observed during phenological stages. The reduction of moderately imbalanced areas and the increase of coordinated areas in SOS and POS stages suggest the improvement of NDVI–EWD coupling, especially in the POS stage, in which the number of moderately coordinated counties increased significantly. In recent decades, Xinjiang has experienced a warming and wetting trend, which is in line with global and regional climate change (Piao et al., 2019; Wang et al., 2020). Better hydrothermal conditions may have relieved drought stress and to some extent promoted vegetation response. At the same time, ecological restoration projects such as the Natural Forest Protection Program, Grain for Green Program, grazing exclusion and grassland restoration projects, and integrated management of the Tarim River Basin have contributed to vegetation recovery in local and key ecological function zones (Jiao et al., 2022; Yuan et al., 2025). The increased coordination during the POS stage resulting from the combined effects of improved hydrothermal conditions and human intervention should not be simply interpreted as an increase in NDVI, but as a coordinated adjustment between increased vegetation productivity and ecological water consumption. The improvement of coordination in the EOS stage was limited, and localized degradation was also observed. This could be associated with lesser water replenishment in autumn, the progressive depletion of soil water, and the seasonal decline in vegetation activity (Wang et al., 2025; Zhang et al., 2025). These results suggest that the enhancement in NDVI-EWD coupling is very seasonal and is limited by the water supply and EWD balance. In Xinjiang, the evidence of a recent wet-to-dry transition and the decreasing soil moisture further supports the view that late-season vegetation-water coupling may be limited by the availability of soil water (Yao et al., 2021).

The quadrant analysis also identified the dynamic evolution of several NDVI–EWD connection types. The long-term dominance of the LL type reflects the core ecological situation of the hinterlands of Xinjiang deserts, where the vegetation cover and the EWD are minimal. However, the LL type area dropped in all three phenological stages from 2000 to 2024, while the HH and HL types increased. The major conversion mechanism is LL to HL transition. This change was mostly in the mountains, river valleys and at the edge of oases. This may be related to the increased vegetation greening resulting from regional warming and wetness, ecological restoration efforts, decreased evapotranspiration demand in high-elevation situations (Ablikim et al., 2023) and soil moisture restrictions. It has been evidenced in previous research that the geographical variability of evapotranspiration in arid environments may prevent the rise of water consumption in some high-altitude or water-scarce regions (Zhou et al., 2022), therefore the increase in vegetation cover may not be connected with high EWD in these places. On the other hand, for the POS stage, a major HL to HH transition means that some oasis agricultural areas, irrigated cropland and river valleys have been changed to a coupled state of high NDVI and high EWD under the circumstance of relatively high-water availability and management inputs. The changes in the vegetation patterns in the wetlands and croplands in the lower Tarim River Basin have also been verified in previous research to be strongly influenced by the ecological water conveyance and irrigation inputs (Wang et al., 2021; Jiao et al., 2022). This is consistent with the distribution of HH kinds along the river corridors and in the oases of southern Xinjiang. The unusual LH to LL transition in the EOS stage implies that some low vegetation cover regions with relatively high EWD changed back into a low-consumption condition due to vegetation senescence or decrease in water supply. This change may be associated to agricultural harvest decreasing plant cover and seasonal reduction in evapotranspiration demand. These patterns show positive benefits of vegetation recovery and ecological restoration initiatives, and also hint at the potential water stress in high water-consuming states during the peak growth season, which can reflect the changeover of quadrant kinds.

### 4.3 Driving mechanisms and their impacts on NDVI–EWD coupling

The formation of the NDVI–EWD coupling pattern was shaped by the combined effects of hydrothermal conditions, terrain-driven water redistribution, soil background, and land-use processes. These driving factors were selected to represent the main ecohydrological controls on vegetation growth and ecological water demand in arid Xinjiang. ET, TMP, and PRE together characterized the regional hydrothermal environment. In particular, ET is directly associated with vegetation transpiration, soil evaporation and regional water use, while TMP controls vegetation phenology, growth activity and atmospheric evaporative demand. PRE is the main water input source and the dominant limiting factor of vegetation growth in arid regions (Zhang et al., 2024b; Lu et al., 2025). TWI demonstrated the terrain’s ability to influence water accumulation and its spatial redistribution, which is especially important for the mountain–oasis–desert gradient in Xinjiang. TWI has also been proven to be a useful topographic proxy for soil moisture and water accumulation processes in the past, but its performance may vary depending on the flow-routing algorithms and the DEM resolution employed (Kopecký et al., 2021; Riihimäki et al., 2021; Winzeler et al., 2022). SOC mainly reflected soil nutrient supply and water-retention capacity. In dryland ecosystems, higher SOC can improve soil structure, nutrient availability, and soil water-holding capacity, thus providing support to vegetation and relieving negative effects of water stress (Rawls et al., 2003; Ramírez et al., 2023). LUI showed high explanatory power in different phenological stages, indicating that the influence of human activities on NDVI–EWD coupling was mainly expressed through land-use processes rather than socioeconomic intensity alone. In Xinjiang, changes in LUI are closely associated with oasis development, cropland expansion, grazing pressure, ecological restoration, irrigation management, and land-cover change. These processes can directly change vegetation cover and indirectly alter ecological water consumption. On the other hand, the relatively weak direct explanatory effects of socioeconomic indicators such as GDP, POP and NTL suggest that human impacts on NDVI–EWD coupling were more strongly associated with land-use patterns than socioeconomic variables themselves. Previous studies have shown that land-use change, irrigation demand and landscape pattern change can have great impacts on water consumption and ecological water requirements in arid and semi-arid areas (Zhao and Lao, 2015; Zhao et al., 2019; Liu et al., 2024). Overall, these results indicate that NDVI–EWD coupling in Xinjiang was not controlled by a single climatic or human activity factor. Instead, it reflected the compound regulation of hydrothermal conditions, terrain-controlled water redistribution, soil properties, and land-use processes. Hydrothermal conditions provided the basic environmental background, terrain controlled the spatial redistribution of water, soil conditions regulated vegetation growth and water retention, and land-use processes further modified the coupling intensity and spatial pattern at the regional scale.

### 4.4 Implications for ecological restoration and water-resource regulation in Xinjiang

Ecological management in arid Xinjiang should not evaluate restoration outcomes based only on vegetation greening. Instead, vegetation improvement, changes in EWD, and regional water resource carrying capacity should be incorporated into a unified assessment framework. Different NDVI–EWD coupling types correspond to different management priorities. For HH areas, vegetation conditions are generally favorable, but EWD is high. Greater attention should therefore be given to ecological water-source security, irrigation efficiency, and the control of water-use intensity. HL areas represent a relatively favorable vegetation–water matching state. These areas should be protected from unreasonable development that may shift them toward a high water-consumption state. For LH areas, insufficient vegetation response or low water-use efficiency may be present. Further diagnosis should be conducted by considering soil conditions, groundwater depth, and land-use structure. Because the POS stage is the period when EWD increases and coupling-type reorganization are most concentrated, monitoring of EWD, optimization of irrigation schedules, and ecological water-supplementation planning should focus on the peak growing season. This is particularly important in areas where vegetation improvement is evident and water demand is high, such as the northern and southern slopes of the Tianshan Mountains, the Ili River Valley, the margins of the Tarim Basin, and the northern foothills of the Kunlun Mountains. In these regions, ecological restoration should be coordinated with agricultural water saving, oasis boundary control, and water-resource allocation. Such coordination is needed to prevent vegetation expansion from exceeding the regional water-resource carrying capacity.

### 4.5 Research limitations and prospects

Although this study revealed the spatiotemporal evolution and driving mechanisms of NDVI–EWD coupling in Xinjiang, several uncertainties remain. The estimation of EWD depends on remote sensing retrievals and model parameters. The accuracy of the results may be influenced by the parameter setting of Kc, Ks, vegetation height and soil moisture limitation. Therefore, in the future studies, flux tower observations, plot scale water consumption experiments, irrigation water use statistics and groundwater monitoring data should be used to validate the model and optimize the parameters. NDVI can detect the variation of vegetation greenness. However, it is subject to saturation in areas with dense vegetation cover. It also has limited ability to distinguish water-consumption differences among natural vegetation, planted forests, and cropland crops. Future work could integrate solar-induced chlorophyll fluorescence (SIF), leaf area index (LAI), actual evapotranspiration products, and crop-type data to improve the identification of EWD from the perspectives of vegetation function and vegetation type. In addition, EWD reflects the potential or relative ecological water-consumption demand of vegetation. It is not equivalent to the actual amount of water available. Thus, future studies should consider water-resource variables, including runoff, groundwater, soil moisture, and irrigation water use, which will help clarify the constraint relationships between vegetation restoration, growing EWD, and regional water-resource carrying capacity.

## 5 Conclusion

This study integrated multi-source remote sensing, climate, soil, topographic, and human activity data from 2000 to 2024 to construct an NDVI–EWD coupling framework for arid Xinjiang across different phenological stages. The main conclusions are as follows:

(1) Vegetation conditions in Xinjiang generally improved from 2000 to 2024, but this greening process was accompanied by an increase in EWD. Therefore, vegetation greening should not be interpreted simply as ecological optimization in arid regions. The POS stage had the highest NDVI and contributed the largest share of EWD, indicating that the peak growing season is the key period for identifying ecological water stress.
(2) The NDVI–EWD coupling coordination degree generally shifted from imbalance toward coordination. Highly coordinated areas were mainly distributed in mountainous regions, river valleys, and oasis agricultural areas, whereas imbalanced areas remained concentrated in the interiors of desert basins. This pattern indicates that the vegetation–water relationship in Xinjiang is strongly constrained by the mountain–oasis–desert gradient.
(3) The quadrant analysis showed that LL remained the dominant coupling type, but its area continued to decrease, while HH and HL types gradually expanded. The LL-to-HL transition was the main conversion pathway, suggesting that vegetation improvement in some areas did not necessarily lead to high ecological water-consumption pressure. However, the HL-to-HH transition during the POS stage indicates that some local areas may face water-resource constraints caused by the coexistence of high vegetation cover and high EWD.
(4) The driving mechanism analysis showed that NDVI–EWD coupling was jointly regulated by ET, LUI, TWI, TMP, PRE, and SOC rather than by a single climatic or human activity factor. These factors reflect the combined effects of hydrothermal conditions, land-use processes, terrain-controlled water redistribution, and soil background on vegetation–water relationships in arid regions.

In summary, this study demonstrates that ecological restoration in arid regions should not be evaluated only by vegetation greening. Changes in EWD and regional water-resource carrying capacity should also be incorporated into an integrated assessment framework to support sustainable vegetation restoration and water-resource management.

## Data Availability

The original contributions presented in the study are included in the article/supplementary material. Further inquiries can be directed to the corresponding author.

## References

[B1] Ablikim K. Yang H. Mamattursun A. (2023). Spatiotemporal variation of evapotranspiration and its driving factors in the Urumqi River Basin. Sustainability 15, 13904. doi: 10.3390/su151813904 30654563

[B2] Allen R. G. Pereira L. S. (2009). Estimating crop coefficients from fraction of ground cover and height. Irrig. Sci. 28, 17–34. doi: 10.1007/s00271-009-0182-z 30311153

[B3] Allen R. G. Pereira L. S. Raes D. Smith M. (1998). Crop evapotranspiration: Guidelines for computing crop water requirements - FAO Irrigation and Drainage Paper 56. (Rome: Food and Agriculture Organization of the United Nations).

[B4] Becker J.-M. Cheah J.-H. Gholamzade R. Ringle C. M. Sarstedt M. (2022). PLS-SEM’s most wanted guidance. Int. J. Contemp. Hosp. M. 35, 321–346. doi: 10.1108/IJCHM-04-2022-0474 35579975

[B5] Chi D. Wang H. Li X. Liu H. Li X. (2018). Estimation of the ecological water requirement for natural vegetation in the Ergune River basin in Northeastern China from 2001 to 2014. Ecol. Indic. 92, 141–150. doi: 10.1016/j.ecolind.2017.04.014 38826717

[B6] D’Odorico P. Scanlon T. M. Runyan C. W. Abshire K. Barrett P. Coloso J. J. . (2009). Dryland ecohydrology: research perspectives. Ann. Arid Zone. 48 (3–4), 229–257. doi: 10.56093/aaz.v48i3

[B7] de Araujo Barbosa C. C. Atkinson P. M. Dearing J. A. (2015). Remote sensing of ecosystem services: A systematic review. Ecol. Indic. 52, 430–443. doi: 10.1016/j.ecolind.2015.01.007 38826717

[B8] Dong S. Zheng J. Li Y. Li Z. Li F. Jin L. . (2019). Quantitative analysis of the coupling coordination degree between urbanization and eco-environment in Mongolia. Chin. Geogr. Sci. 29, 861–871. doi: 10.1007/s11769-019-1074-7 30311153

[B9] Fan Q. Lu Q. Liu B. Li J. Ping X. Yang X. (2025). Research on the centre of gravity after “Transfer-Conversion-Changes” in different land use types during 2010–2020. Sci. Rep. 15, 21722. doi: 10.1038/s41598-025-06185-5 40594949 PMC12216847

[B10] Fan M. Xu J. Chen Y. Li D. Tian S. (2020). How to sustainably use water resources—a case study for decision support on the water utilization of Xinjiang, China. Water 12, 3564. doi: 10.3390/w12123564 30654563

[B11] Fu A. Li W. Wang Y. (2021). Calculation of targeted eco-environmental water requirements in a dry inland river: a case study of the Yarkand River Basin, Xinjiang, China. SN Appl. Sci. 3, 680. doi: 10.1007/s42452-021-04676-4 30311153

[B12] Gann D. (2019). Quantitative spatial upscaling of categorical information: The multi-dimensional grid-point scaling algorithm. Methods Ecol. Evol. 10, 2090–2104. doi: 10.1111/2041-210X.13301 40046247

[B13] Gao D. (2024). Study on the coupling coordination and barrier factors between agroecological security and rural green development in China. Sci. Rep. 14, 29767. doi: 10.1038/s41598-024-80669-8 39613808 PMC11606961

[B14] Ge Z. Huang J. Wang X. Zhao Y. Tang X. Zhou Y. . (2021). Using remote sensing to identify the peak of the growing season at globally-distributed flux sites: A comparison of models, sensors, and biomes. Agric. For. Meteorol. 307, 108489. doi: 10.1016/j.agrformet.2021.108489 38826717

[B15] Guo J. Wei X. Zhang F. Ding Y. (2025). Coupled assessment of land use changes and ecological benefits using multi-source remote sensing data. Agriculture-london. 15, 1358. doi: 10.3390/agriculture15131358 30654563

[B16] Hair J. F. Jr. Hult G. T. M. Ringle C. M. Sarstedt M. Danks N. P. Ray S. (2021). Partial Least Squares Structural Equation Modeling (Pls-Sem) Using R: A Workbook. (Cham: Springer Nature). doi: 10.1007/978-3-030-80519-7

[B17] Hao X. Zhao Z. Fan X. Zhang J. Zhang S. (2023). Evaluation method of ecological water demand threshold of natural vegetation in arid-region inland river basin based on satellite data. Ecol. Indic. 146, 109811. doi: 10.1016/j.ecolind.2022.109811 38826717

[B18] He X. Liu W. (2024). Analysis of spatial patterns and influencing factors of farmland transfer in China based on ESDA-GeoDetector. Sci. Rep. 14, 12485. doi: 10.1038/s41598-024-62931-1 38816491 PMC11139945

[B19] Higgins S. I. Conradi T. Muhoko E. (2023). Shifts in vegetation activity of terrestrial ecosystems attributable to climate trends. Nat. Geosci. 16, 147–153. doi: 10.1038/s41561-022-01114-x 37880705

[B20] Huang M. Mu Z. Zhao S. Yang R. (2024). Ecological water requirement of natural vegetation in the Tarim River Basin based on multi-source data. Sustainability 16, 7034. doi: 10.3390/su16167034 30654563

[B21] Huang S. Tang L. Hupy J. P. Wang Y. Shao G. (2020). A commentary review on the use of normalized difference vegetation index (NDVI) in the era of popular remote sensing. J. For. Res. 32, 1–6. doi: 10.1007/s11676-020-01155-1 30311153

[B22] Jia Z. Lin T. Guo X. Zheng Y. Geng H. Zhang J. . (2024). Vegetation greening mitigates the positive impacts of climate change on water availability in Northwest China. J. Hydrol. 644, 132086. doi: 10.1016/j.jhydrol.2024.132086 38826717

[B23] Jiao A. Wang W. Ling H. Deng X. Yan J. Chen F. (2022). Effect evaluation of ecological water conveyance in Tarim River Basin, China. Front. Environ. Sci. 10. doi: 10.3389/fenvs.2022.1019695

[B24] Kang S. Jia X. Zhao Y. Luo M. Wang H. Zhao M. (2024). The coupling coordination relationship between urbanization and the eco-environment in resource-based cities, Loess Plateau, China. ISPRS Int. J. Geo-Inf. 13, 437. doi: 10.3390/ijgi13120437 30654563

[B25] Ke S. Cui H. Lu X. (2025). GeoDetector analysis of spatio-temporal patterns and driving mechanisms of farmland spatial transition within 897 counties in the Yangtze River Economic Belt, China. Humanit. Soc Sci. Commun. 12, 923. doi: 10.1057/s41599-025-05354-1

[B26] Kooistra L. Berger K. Brede B. Graf L. V. Aasen H. Roujean J.-L. . (2024). Reviews and syntheses: Remotely sensed optical time series for monitoring vegetation productivity. Biogeosciences 21, 473–511. doi: 10.5194/bg-21-473-2024

[B27] Kopecký M. Macek M. Wild J. (2021). Topographic Wetness Index calculation guidelines based on measured soil moisture and plant species composition. Sci. Total Environ. 757, 143785. doi: 10.1016/j.scitotenv.2020.143785 33220998

[B28] Kou L. Wang X. Wang H. Wang X. Hou Y. (2024). Spatiotemporal analysis of ecological benefits coupling remote sensing ecological index and ecosystem services index. Ecol. Indic. 166, 112420. doi: 10.1016/j.ecolind.2024.112420 38826717

[B29] Li J. Sun W. Li M. Meng L. (2021). Coupling coordination degree of production, living and ecological spaces and its influencing factors in the Yellow River Basin. J. Cleaner Prod. 298, 126803. doi: 10.1016/j.jclepro.2021.126803 38826717

[B30] Li Z.-L. Wu H. Duan S.-B. Zhao W. Ren H. Liu X. . (2023). Satellite remote sensing of global land surface temperature: Definition, methods, products, and applications. Rev. Geophys. 61, e2022RG000777. doi: 10.1029/2022RG000777 29143083

[B31] Ling H. Guo B. Xu H. Fu J. (2014). Configuration of water resources for a typical river basin in an arid region of China based on the ecological water requirements (EWRs) of desert riparian vegetation. Global Planet. Change 122, 292–304. doi: 10.1016/j.gloplacha.2014.09.008 38826717

[B32] Liu H. Lu J. Li X. Wang Y. Xu D. Yin J. . (2025). Evaluating human-nature relationships at a grid scale in China 2000–2020. Habitat Int. 156, 103282. doi: 10.1016/j.habitatint.2024.103282 38826717

[B33] Liu X. Tang Q. Zhao Y. Wang P. (2024). Persistent water scarcity due to high irrigation demand in arid China: A case study in the North Slope of the Tianshan Mountains. Earths Future 12, e2024EF005070. doi: 10.1029/2024EF005070 29143083

[B34] Liu Y. Wu C. Wang X. Jassal R. S. Gonsamo A. (2021). Impacts of global change on peak vegetation growth and its timing in terrestrial ecosystems of the continental US. Global Planet. Change 207, 103657. doi: 10.1016/j.gloplacha.2021.103657 38826717

[B35] Lu Y. Yu Y. Sun L. Li C. He J. Guo Z. . (2025). NDVI based vegetation dynamics and responses to climate change and human activities at Xinjiang from 2001 to 2020. Sci. Rep. 15, 25848. doi: 10.1038/s41598-025-11677-5 40670565 PMC12267569

[B36] Mann H. B. (1945). Nonparametric tests against trend. Econometrica 13, 245. doi: 10.2307/1907187

[B37] Mehmood K. Anees S. A. Rehman A. Pan S. Tariq A. Zubair M. . (2024). Exploring spatiotemporal dynamics of NDVI and climate-driven responses in ecosystems: Insights for sustainable management and climate resilience. Ecol. Inf. 80, 102532. doi: 10.1016/j.ecoinf.2024.102532 38826717

[B38] Mekonnen Y. G. Alamirew T. Chukalla A. D. Malede D. A. Yalew S. G. Mengistu A. F. (2025). Remote sensing in hydrology: A systematic review of its applications in the Upper Blue Nile Basin, Ethiopia. HydroResearch 8, 1–12. doi: 10.1016/j.hydres.2024.09.002 38826717

[B39] Miralles D. G. Bonte O. Koppa A. Baez-Villanueva O. M. Tronquo E. Zhong F. . (2025). GLEAM4: global land evaporation and soil moisture dataset at 0.1° resolution from 1980 to near present. Sci. Data 12, 416. doi: 10.1038/s41597-025-04610-y 40064907 PMC11894173

[B40] Mo K. Chen Q. Chen C. Zhang J. Wang L. Bao Z. (2019). Spatiotemporal variation of correlation between vegetation cover and precipitation in an arid mountain-oasis river basin in northwest China. J. Hydrol. 574, 138–147. doi: 10.1016/j.jhydrol.2019.04.044 38826717

[B41] Parmesan C. Morecroft M. D. Trisurat Y. Mezzi D. (2022). “ Terrestrial and Freshwater Ecosystems and Their Services.” in Climate Change 2022: Impacts, Adaptation and Vulnerability. Contribution of Working Group II to the Sixth Assessment Report of the Intergovernmental Panel on Climate Change, eds. Pörtner H.-O. Roberts D. C. (UK and USA: Cambridge University Press and Princeton University Press). doi: 10.1017/9781009325844.004

[B42] Pereira L. S. Paredes P. Espírito-Santo D. (2024). Crop coefficients of natural wetlands and riparian vegetation to compute ecosystem evapotranspiration and the water balance. Irrig. Sci. 42, 1171–1197. doi: 10.1007/s00271-024-00923-9 30311153

[B43] Piao S. Wang X. Park T. Chen C. Lian X. He Y. . (2019). Characteristics, drivers and feedbacks of global greening. Nat. Rev. Earth Environ. 1, 14–27. doi: 10.1038/s43017-019-0001-x 37880705

[B44] Ramírez P. B. MaChado S. Singh S. Plunkett R. Calderón F. J. (2023). Addressing the effects of soil organic carbon on water retention in US Pacific Northwest wheat–soil systems. Front. Soil Sci. 3. doi: 10.3389/fsoil.2023.1233886

[B45] Rawls W. J. Pachepsky Y. A. Ritchie J. C. Sobecki T. M. Bloodworth H. (2003). Effect of soil organic carbon on soil water retention. Geoderma 116, 61–76. doi: 10.1016/S0016-7061(03)00094-6

[B46] Riihimäki H. Kemppinen J. Kopecký M. Luoto M. (2021). Topographic Wetness Index as a proxy for soil moisture: The importance of flow-routing algorithm and grid resolution. Water Resour. Res. 57, e2021WR029871. doi: 10.1029/2021WR029871 29143083

[B47] Ringle C. M. Sarstedt M. Sinkovics N. Sinkovics R. R. (2023). A perspective on using partial least squares structural equation modelling in data articles. Data Brief 48, 109074. doi: 10.1016/j.dib.2023.109074 37066088 PMC10090253

[B48] Sandholt I. Rasmussen K. Andersen J. (2002). A simple interpretation of the surface temperature/vegetation index space for assessment of surface moisture status. Remote Sens. Environ. 79, 213–224. doi: 10.1016/S0034-4257(01)00274-7

[B49] Sen P. K. (1968). Estimates of the regression coefficient based on Kendall’s Tau. J. Am. Stat. Assoc. 63, 1379–1389. doi: 10.1080/01621459.1968.10480934 37339054

[B50] Sun J. Ding Y. Wang Y. E C. (2025). Vegetation dynamics and its driving force in the Qinghai Lake Basin, China. Front. Plant Sci. 16. doi: 10.3389/fpls.2025.1691672 41195140 PMC12583180

[B51] Sun Y. Liu S. Shi F. An Y. Li M. Yixuan L. (2020). Spatio-temporal variations and coupling of human activity intensity and ecosystem services based on the four-quadrant model on the Qinghai-Tibet Plateau. Sci. Total Environ. 743, 140721. doi: 10.1016/j.scitotenv.2020.140721 32679497

[B52] Wang Z. Fu B. Wu X. Li Y. Feng Y. Wang S. . (2023c). Vegetation resilience does not increase consistently with greening in China’s Loess Plateau. Commun. Earth Environ. 4, 336. doi: 10.1038/s43247-023-01000-3 37880705

[B53] Wang H. Liu Y. Wang Y. Yao Y. Wang C. (2023a). Land cover change in global drylands: A review. Sci. Total Environ. 863, 160943. doi: 10.1016/j.scitotenv.2022.160943 36526201

[B54] Wang Y. Wu Y. Zhao S. Wang G. (2025). Long-term response of soil moisture to vegetation changes in the drylands of Northern China. Sustainability 17, 2483. doi: 10.3390/su17062483 30654563

[B55] Wang R. Zayit A. He X. Han D. Yang G. Lv G. (2023b). Ecological water requirement of vegetation and water stress assessment in the middle reaches of the Keriya River Basin. Remote Sens. 15, 4638. doi: 10.3390/rs15184638 30654563

[B56] Wang Q. Zhai P.-M. Qin D.-H. (2020). New perspectives on ‘warming–wetting’ trend in Xinjiang, China. Adv. Clim. Change Res. 11, 252–260. doi: 10.1016/j.accre.2020.09.004 38826717

[B57] Wang J.-F. Zhang T.-L. Fu B.-J. (2016). A measure of spatial stratified heterogeneity. Ecol. Indic. 67, 250–256. doi: 10.1016/j.ecolind.2016.02.052 38826717

[B58] Wang S. Zhou K. Zuo Q. Wang J. Wang W. (2021). Land use/land cover change responses to ecological water conveyance in the lower reaches of Tarim River, China. J. Arid. Land 13, 1274–1286. doi: 10.1007/s40333-021-0089-y 30311153

[B59] Winzeler H. E. Owens P. R. Read Q. D. Libohova Z. Ashworth A. Sauer T. (2022). Topographic Wetness Index as a proxy for soil moisture in a hillslope catena: Flow algorithms and map generalization. Land 11, 2018. doi: 10.3390/land11112018 30654563

[B60] Xia N. Tang Y. Tang M. Quan W. Xu Z. Zhang B. . (2024). Monitoring and evaluation of vegetation restoration in the Ebinur Lake Wetland National Nature Reserve under lockdown protection. Front. Plant Sci. 15. doi: 10.3389/fpls.2024.1332788 38699539 PMC11063322

[B61] Xiong Y. Zhang Y. Zhang Z. Feng T. Wang P. Keesstra S. (2024). The effects and contributions of ecological factors on soil carbon, water and nutrient storages under long-term vegetation restoration on the eastern Loess Plateau. Forests 15, 1898. doi: 10.3390/f15111898 30654563

[B62] Yang H. Wang L. Tang F. Fu M. Xiong Y. (2024). Differences in urban vibrancy enhancement among different mixed land use types: Evidence from Shenzhen, China. Land 13, 1661. doi: 10.3390/land13101661 30654563

[B63] Yao J. Chen Y. Guan X. Zhao Y. Chen J. Mao W. (2022). Recent climate and hydrological changes in a mountain–basin system in Xinjiang, China. Earth Sci. Rev. 226, 103957. doi: 10.1016/j.earscirev.2022.103957 38826717

[B64] Yao J. Mao W. Chen J. Dilinuer T. (2021). Recent signal and impact of wet-to-dry climatic shift in Xinjiang, China. J. Geogr. Sci. 31, 1283–1298. doi: 10.1007/s11442-021-1898-9 30311153

[B65] Yuan H. Yang R. Wu J. Zhang J. Zhou L. (2025). Vegetation productivity in Xinjiang’s ecosystems: Responses to natural and human factors across basins and land covers. Ecosyst. Health Sustainability 11, 338. doi: 10.34133/ehs.0338

[B66] Zhang Q. Gu L. Liu Y. Zhang Y. (2024b). Spatio-temporal dynamics of normalized difference vegetation index and its response to climate change in Xinjiang 2000–2022. Forests 15, 370. doi: 10.3390/f15020370 30654563

[B67] Zhang H. Hu Z. Zhang Z. Li Y. Song S. Chen X. (2024a). How does vegetation change under the warm–wet tendency across Xinjiang, China? Int. J. Appl. Earth Obs. Geoinf. 127, 103664. doi: 10.1016/j.jag.2024.103664 38826717

[B68] Zhang X. Li X. Wang Z. Liu Y. Yao L. (2024c). A study on matching supply and demand of ecosystem services in the Hexi region of China based on multi-source data. Sci. Rep. 14, 1332. doi: 10.1038/s41598-024-51805-1 38228656 PMC10791686

[B69] Zhang W. Li Y. Wu X. Chen Y. Chen A. Schwalm C. R. . (2021). Divergent response of vegetation growth to soil water availability in dry and wet periods over Central Asia. J. Geophys. Res. Biogeosci. 126, e2020JG005912. doi: 10.1029/2020JG005912 29143083

[B70] Zhang X. Wang X. Zohner C. M. Peñuelas J. Li Y. Wu X. . (2025). Declining precipitation frequency may drive earlier leaf senescence by intensifying drought stress and enhancing drought acclimation. Nat. Commun. 16, 910. doi: 10.1038/s41467-025-56159-4 39837832 PMC11750966

[B71] Zhang Y. Zhang J. Xia J. Guo Y. Fu Y. H. (2022). Effects of vegetation phenology on ecosystem water use efficiency in a semiarid region of Northern China. Front. Plant Sci. 13. doi: 10.3389/fpls.2022.945582 35860533 PMC9289613

[B72] Zhang M. Zhou Y. Li X. Sun Z. Yang G. Xie Z. (2023). Quantifying the contributions of regional human activities and global climate change to the regional climate in a typical mountain-oasis-desert system of arid Central Asia from 1979 to 2018. J. Geophys. Res. Atmos. 128, e2022JD037110. doi: 10.1029/2022JD037110 29143083

[B73] Zhao H. Lao R. (2015). Change in water consumption and its effect on the land cover of the oasis in the Tarim River Basin, Xinjiang, China. Nat. Environ. pollut. Technol. 14, 1011–1018.

[B74] Zhao F. Li H. Li C. Cai Y. Wang X. Liu Q. (2019). Analyzing the influence of landscape pattern change on ecological water requirements in an arid/semiarid region of China. J. Hydrol. 578, 124098. doi: 10.1016/j.jhydrol.2019.124098 38826717

[B75] Zhou Y. Li Y. Li W. Li F. Xin Q. (2022). Ecological responses to climate change and human activities in the arid and semi-arid regions of Xinjiang in China. Remote Sens. 14, 3911. doi: 10.3390/rs14163911 30654563

[B76] Zhu Y. Zheng Z. Zhao G. Zhu J. Zhao B. Sun Y. . (2025). Evapotranspiration increase is more sensitive to vegetation greening than to vegetation type conversion in arid and semi-arid regions of China. Global Planet. Change 244, 104634. doi: 10.1016/j.gloplacha.2024.104634 38826717

